# Assessing the utility of epigenetic clocks for health prediction in South Korean

**DOI:** 10.3389/fragi.2024.1493406

**Published:** 2024-12-02

**Authors:** Dong Jun Kim, Joon Ho Kang, Ji-Woong Kim, Sun bin Kim, Young Kee Lee, Myeong Jae Cheon, Byung-Chul Lee

**Affiliations:** Genoplan Korea, Seoul, Republic of Korea

**Keywords:** epigenetic clock, lifestyle, methylation, healthcare, aging

## Abstract

Epigenetic clocks have been developed to track both chronological age and biological age, which is defined by physiological biomarkers and the risk of adverse health outcomes. Epigenetic age acceleration (EAA) has been found to predict various diseases, aging-related factors, and mortality. However, epigenetic clocks have predominantly been developed with individuals of European or Hispanic ancestry, and their association with health outcomes and environmental factors has not been sufficiently assessed in East Asian populations. Here, we investigated nine epigenetic clocks: five trained on chronological age (first-generation) and four on biological age (second-generation), using DNA methylation data from blood samples of South Koreans. EAAs of second-generation epigenetic clocks reflected the risk of chronic diseases (type 2 diabetes and hypertension), levels of health-related blood markers (alanine aminotransferase, aspartate aminotransferase, high density lipoprotein, triglyceride, and high sensitivity C-reactive protein), and lung functions (percentage of predicted FEV1 and percentage of predicted FVC), while EAAs of first generation clocks did not. Using follow-up data, we also found that EAAs of second-generation clocks were associated with the time to onset risks of chronic diseases. Health behavior factors (drinking, smoking, exercise, body mass index, and waist-hip ratio), socioeconomic status (income level and educational attainment), and psychosocial status were associated with EAAs of second-generation clocks, while only smoking status was associated with EAAs of first-generation clocks. We conducted validation analyses in an independent South Korean cohort and replicated the association of EAAs with health outcomes and environmental factors. Age acceleration of epigenetic clocks is influenced by various environmental factors and can serve as an effective predictor of health in South Korea.

## Introduction

Over the past decade, there has been a substantial increase in the development and application of epigenetic predictors in healthcare research (Yousefi et al., 2022). DNA methylation changes dynamically in response to a variety of exogenous and endogenous factors, and can be used to estimate age through predictors called “epigenetic clocks” (Horvath and Raj, 2018). Epigenetic age acceleration (EAA), typically derived by regressing the epigenetic age of clocks on chronological age, assesses whether individuals are aging faster or slower than their chronological age. EAA provides valuable insights into health outcomes, including morbidity and mortality (Horvath and Raj, 2018; Jain et al., 2022), and has been linked to health behaviors such as body mass index (BMI), alcohol consumption, smoking, and physical activity (Quach et al., 2017; Ryan et al., 2020).

First-generation epigenetic clocks, exemplified by Horvath’s clock and Hannum’s clock (Horvath, 2013; Hannum et al., 2013), were trained on chronological age and used to estimate chronological age (Horvath, 2013; Hannum et al., 2013; Bernabeu et al., 2023; Zhang et al., 2019). Second-generation epigenetic clocks have been developed to track biological age based on diverse age-related metrics, encompassing clinical biomarkers (PhenoAge) (Levine et al., 2018), pace of aging (DunedinPACE) (Belsky et al., 2022), and all-cause mortality (GrimAge and Zhang Y’s clock) (Lu et al., 2019; Zhang et al., 2017). Because they focus on different parts of aging, “second-generation” clocks outperform “first-generation” clocks in predicting both life span and health span (Zhang et al., 2019; Levine et al., 2018; Bell et al., 2019; Faul et al., 2023). Recently, principal component (PC)-based clocks have been created to mitigate noise and unreliability from individual CpG sites using PCs (Higgins-Chen et al., 2022).

With the increasing number of epigenetic clocks, various studies have compared these clocks with each other and have identified associations with health indicators and mortality. For example, Faul et al. (2023) and Crimmins et al. (2021) utilized epigenetic clocks in US adults and identified that second-generation clocks are linked to health behaviors, morbidity, and mortality. McCrory et al. (2021) and Maddock et al. (2020) demonstrated an association of PhenoAge and GrimAge with health span in British population, while Lu et al. (2019) found a predictive utility of GrimAge for morbidity and mortality in a cohort comprising individuals of European, American, and Hispanic ancestry.

Most epigenetic clocks have been primarily developed using data from European, African, or Hispanic individuals (Horvath, 2013; Hannum et al., 2013; Bernabeu et al., 2023; Zhang et al., 2019; Levine et al., 2018; Belsky et al., 2022; Lu et al., 2019; Zhang et al., 2017). Studies investigating their effects on health outcomes or the influence of environmental factors have also predominantly focused on these ancestry groups (Faul et al., 2023; Crimmins et al., 2021; McCrory et al., 2021; Maddock et al., 2020). However, notable differences exist in epigenetic clocks among various ethnic groups (Crimmins et al., 2021; Horvath et al., 2016), and very few studies have investigated their application in the East Asian population.

To assess the performance of epigenetic clocks in the East Asian population, we examined the association of various epigenetic clocks with health outcomes and environmental factors in 1,925 South Korean samples. We employed five “first-generation” epigenetic clocks [PCHorvath (Horvath et al., 2018), PCHannum (Hannum et al., 2013), ZhangQ (Zhang et al., 2019), Bernabeu (Bernabeu et al., 2023), and iCAS-DNAmAge (Zheng et al., 2024)], along with four “second-generation” epigenetic clocks [PCPhenoAge (Levine et al., 2018), PCGrimAge (Lu et al., 2019), DunedinPACE (Belsky et al., 2022), and ZhangY (Zhang et al., 2017)]. We explored the association of EAAs from each clock with health outcomes, health behaviors, and the time to onset of chronic diseases, and confirmed these findings in an independent South Korean cohort. Our findings highlight the utility of epigenetic clocks in predicting adverse health outcomes in East Asians.

## Results

### Basic characteristics of participants

For the analysis, we used data from Korea Association Resource (KARE) of the Korean Genome and Epidemiology Study (KoGES) (Kim et al., 2017). The basic characteristics of the 1,925 KARE participants are shown in Table 1. The chronological age ranged from 47 to 78 years, with 1,006 (52.3%) males and 919 (47.7%) females. Except for the psychosocial wellbeing index (PWI) and hypertension, significant mean differences in characteristics were observed between males and females (*t*-test *p*-value < 0.05). Females generally exhibited better health across various indicators such as T2D prevalence and blood marker levels compared to males. Females also demonstrated healthier behavior, including a lower waist-hip ratio (WHR), less drinking and smoking, and regular exercise, but their BMI tended to be higher than males.

**TABLE 1:** Basic characteristics of 1,925 KARE participants.

	Males	Females
Total N	1,006 (52.3%)	919 (47.7%)
Chronological age (years)*	59.6 (8.5)	60.5 (9)
Body mass index*	24.1 (2.9)	24.6 (3.2)
Waist-hip ratio*	0.93 (0.06)	0.92 (0.09)
Income level*	4.5 (2.2)	3.5 (2.2)
Educational attainment*	3.2 (1.6)	2.1 (1.4)
PWI	17.2 (8.5)	18 (8.9)
Drinking status*		
Never	235 (23.4%)	705 (76.8%)
Ever	80 (8%)	16 (1.7%)
Current	691 (68.7%)	197 (21.5%)
Smoking status*		
Never	255 (25.3%)	887 (96.6%)
Ever	400 (39.8%)	13 (1.4%)
Current	351 (34.9%)	18 (2%)
Pack-years of smoking*	29 (19.9)	10.6 (10.6)
Regular exercise*	440 (43.7%)	591 (64.3%)
Type 2 diabetes*	332 (33%)	248 (27%)
Hypertension	436 (43.3%)	411 (44.7%)
AST* (IU/L)	28.5 (16.1)	24.1 (10)
ALT* (IU/L)	28.2 (18.3)	21.4 (12.6)
HDL* (mg/dL)	42 (11.2)	44.4 (10.8)
Triglyceride* (mg/dL)	143.2 (77.4)	132.5 (70.6)
hs-CRP* (mg/L)	1.9 (5.2)	1.4 (2.7)
FVC % PRED*	101.3 (13.1)	107.9 (14.9)
FEV1% PRED*	106.4 (15.8)	118.2 (18)

All data are presented as mean ± standard deviation or numbers (%). Missing samples for each value are excluded. * *p*-value of *t*-test between male and female is less than 0.05. PWI, psychosocial wellbeing index; AST, alanine aminotransferase; ALT, aspartate aminotransferase; HDL, high density lipoprotein; hs-CRP, high sensitivity C-reactive protein; FVC % PRED, percentage of predicted FVC; FEV1% PRED, percentage of predicted FEV1.

We calculated epigenetic ages from eight epigenetic clocks, comprising five first-generation clocks [Bernabeu (Bernabeu et al., 2023), iCAS-DNAmAge (Zheng et al., 2024), ZhangQ (Zhang et al., 2019), PCHannum (Hannum et al., 2013), and PCHorvath (Horvath et al., 2018)] and four second-generation clocks [PCGrimAge (Lu et al., 2019), PCPhenoAge (Levine et al., 2018), DunedinPACE (Belsky et al., 2022), and ZhangY (Zhang et al., 2017)]. The accuracy of age prediction was assessed through Pearson correlation analysis between the epigenetic clocks and chronological age (Supplementary Table S1). All epigenetic clocks showed correlations with chronological age, with the Bernabeu clock displaying the highest predictive accuracy (r = 0.96). Although iCAS-DNAmAge was developed in East Asians, it showed a relatively low correlation (r = 0.79). Additionally, the epigenetic ages of each clock were strongly correlated with each other (Supplementary Figure S1). Bernabeu and ZhangQ exhibited the strongest correlation between them (r = 0.98), despite the substantial differences in the CpG sites used.

**FIGURE 1:**
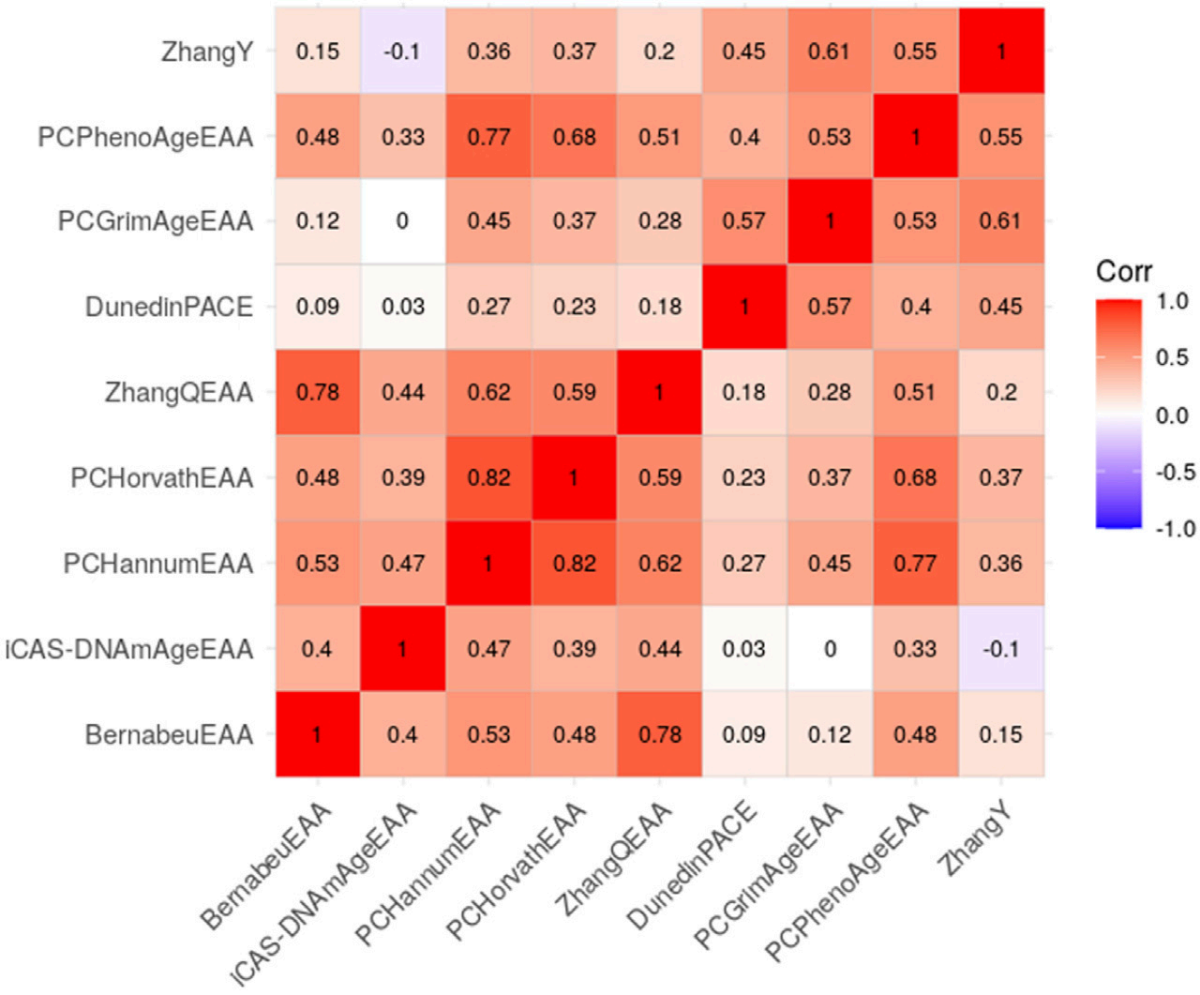
Correlation matrix of epigenetic age accelerations. Positive correlations are denoted by red shades, with lighter shades signifying weaker correlation values.

EAAs of each clock were generated by regressing epigenetic age on chronological age. Except EAA of iCAS-DNAmAge, all EAAs exhibited correlations among themselves (Figure 1), and showing relatively strong correlations (0.39–0.82) observed within the same generation and comparatively weaker correlations (−0.1–0.77) observed between different generations. The strongest correlation was observed between PCHorvathEAA and PCHannumEAA (r = 0.82), while iCAS-DNAmAgeEAA showed the no correlation with PCGrimAgeEAA (r = 0) and a negative correlation with ZhangY. Males exhibited a significantly accelerated aging rate compared to females across all epigenetic clocks, except iCAS-DNAmAge (*t*-test *p*-value < 0.05, Supplementary Table S2).

### Epigenetic age acceleration and health outcomes

To explore which EAAs of epigenetic clocks reflect health outcomes, we conducted regression analyses adjusting for covariates: age, sex, and 10PCs, which were included to account for noise in individual CpG sites. We estimated the effects of EAAs on various health outcomes, including T2D, hypertension, alanine aminotransferase (ALT), aspartate aminotransferase (AST), high density lipoprotein (HDL), triglyceride (TG), and high sensitivity C-reactive protein (hs-CRP), percentage of predicted FEV1 (FEV1% PRED), and percentage of predicted FVC (FVC% PRED). The significance of the association results was determined using a *p*-value threshold adjusted for multiple testing via Bonferroni correction [*p*-value < 0.05/(nine health outcomes × number of clocks) = 1.11E-03 for first-generation clocks and 1.39E-03 for second-generation clocks].

**FIGURE 2:**
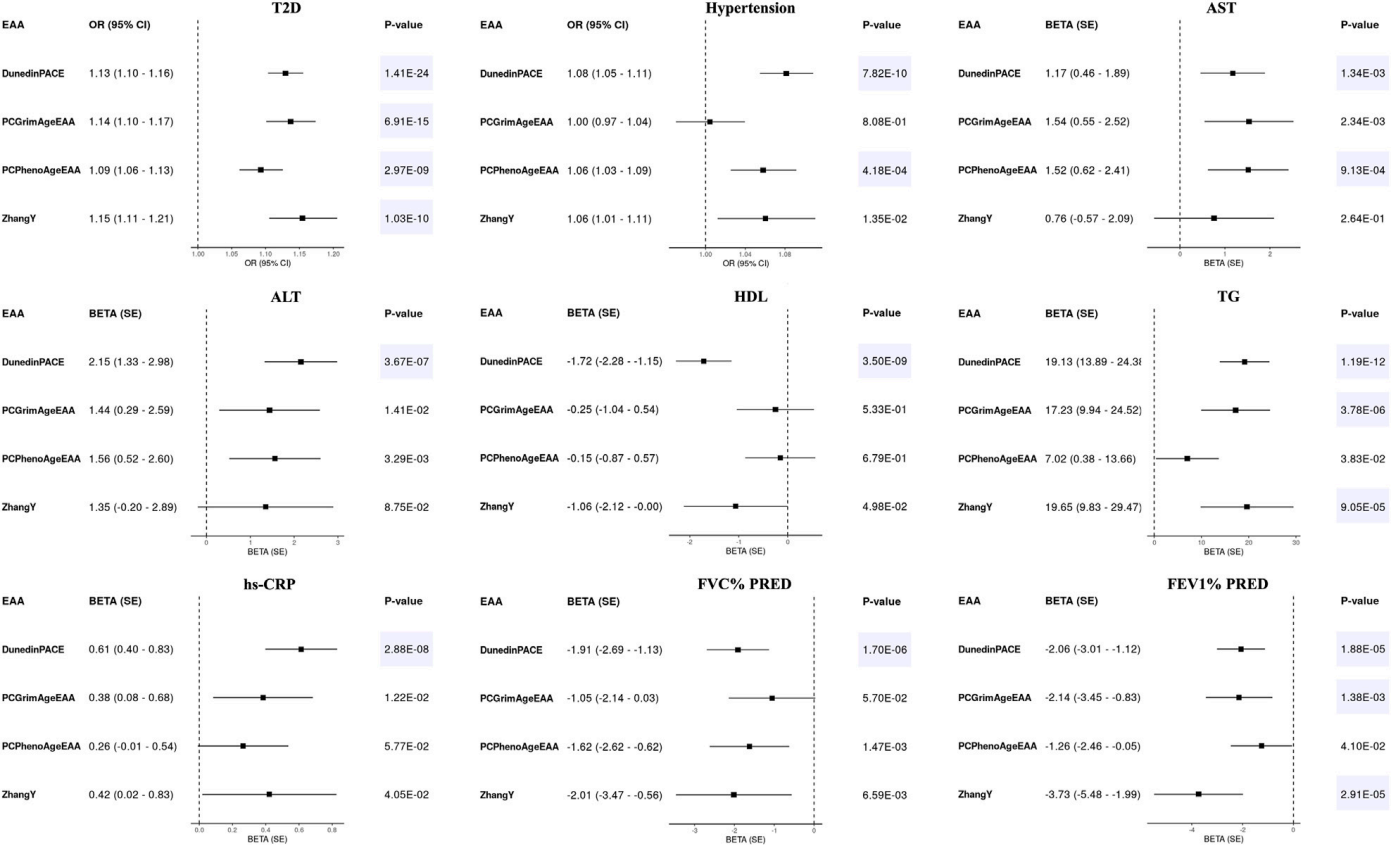
Forest plots for epigenetic age accelerations (EAAs) of second-generation clocks and health outcomes in KARE. Odds ratios (ORs) with 95% confidence intervals (CIs) or beta values with standard errors (SEs) were displayed along with their corresponding *p*-values. All regression models adjusted for age, sex, and 10 principal components, with all EAAs scaled to a mean of 0 and a standard deviation of 1. Significant results were highlighted with a blue background based on Bonferroni-corrected *p*-values [0.05/(4 × 9) = 1.39E-03].

None of the EAAs from the first-generation epigenetic clocks were significantly associated with any diseases or health indicators (*p*-value > 1.11E-03, Supplementary Table S3). Only some EAAs showed a weak positive association with the risk of T2D and hypertension, as well as with an increase in AST levels, satisfying the uncorrected *p*-value threshold [Odds ratio (OR) > 1 or Beta >0, *p*-value < 0.05]. On the other hand, EAAs of the second-generation clocks showed significant associations (*p*-value < 1.39E-03), suggesting that higher EAAs have a detrimental effect on health (Figure 2). The faster aging in all second-generation epigenetic clocks was associated with an increased risk of T2D. In addition, higher DunedinPACE was associated with a higher risk of hypertension (OR > 1), as well as elevated levels of AST, ALT, TG, and hs-CRP (Beta > 0). Conversely, it was associated with decreased levels of HDL, FEV1% PRED, and FVC% PRED (Beta < 0). PCGrimAgeEAA and ZhangY showed a positive association with levels of TG, and a negative association with FEV1% PRED. Higher PCPhenoAgeEAA indicated a significant association with an increased risk of hypertension and elevated AST levels.

Since EAAs of epigenetic clocks were associated with the risk of T2D and hypertension, we further investigated their association with time to onset. Using follow-up data, we conducted analyses with 123 cases of T2D and 721 controls, as well as 277 cases of hypertension and 494 controls. In Kaplan-Meier curves, each EAA was categorized into two groups: age deceleration and age acceleration. DunedinPACE and ZhangY were classified into age acceleration and deceleration groups based on their top 50% and bottom 50% (Supplementary Figure S2). The EAAs from the first-generation epigenetic clocks did not exhibit any association with the onset of T2D and hypertension (*p*-value > 0.05). Among the second-generation clocks, DunedinPACE showed a significant association with the onset of T2D (*p*-value < 0.05/(Two diseases × number of clocks) = 5E-03 for first-generation clocks and 6.25E-03 for second-generation clocks), while PCGrimAgeEAA, DunedinPACE, and ZhangY satisfied uncorrected *p*-value (*p*-value < 0.05) in their association with the onset of hypertension. For Cox regression analysis, EAAs from first-generation clocks did not show an association with the onset of T2D (*p*-value > 6.25E-3, Table 2). iCAS-DNAmAge is significantly associated with the development of hypertension, but its acceleration was associated with a reduced risk of hypertension onset (Hazard ratio < 1). In contrast, higher values of DunedinPACE, PCGrimAgeEAA, and ZhangY were significantly associated with a higher risk of T2D onset (Hazard ratio > 1, *p*-value < 6.25E-03). Increased values of DunedinPACE and ZhangY were associated with an increased risk of hypertension onset as well.

**TABLE 2:** Cox regression analysis of epigenetic age acceleration for onset of type 2 diabetes and hypertension.

		Disease	HR (95% CI)	*p*-value
First-generation	BernabeuEAA	T2D	0.81 (0.66, 0.98)	3.23E-02
		Hypertension	0.89 (0.78, 1.01)	6.52E-02
	iCAS-DNAmAgeEAA	T2D	0.77 (0.59, 1.01)	6.03E-02
		Hypertension	0.78 (0.66, 0.92)	3.32E-03
	PCHannumEAA	T2D	0.75 (0.56, 1.02)	6.87E-02
		Hypertension	0.90 (0.74, 1.1)	3.09E-01
	PCHorvathEAA	T2D	0.96 (0.73, 1.27)	7.84E-01
		Hypertension	0.84 (0.7, 1.01)	6.60E-02
	ZhangQEAA	T2D	0.87 (0.71, 1.07)	1.84E-01
		Hypertension	0.97 (0.84, 1.11)	6.65E-01
Second-generation	DunedinPACE	T2D	1.99 (1.60, 2.48)	7.00E-10
		Hypertension	1.28 (1.12, 1.47)	3.66E-04
	PCGrimAgeEAA	T2D	1.50 (1.14, 1.98)	3.65E-03
		Hypertension	1.06 (0.87, 1.3)	5.36E-01
	PCPhenoAgeEAA	T2D	1.05 (0.81, 1.37)	7.10E-01
		Hypertension	1.02 (0.86, 1.22)	8.11E-01
	ZhangY	T2D	2.34 (1.60, 3.41)	1.06E-05
		Hypertension	1.55 (1.20, 2)	7.18E-04

All Cox models are adjusted for age, sex, and 10 principal components. All EAAs are scaled to mean = 0 and SD = 1. HR, Hazard ratios; CI, confidence interval.

### Epigenetic age acceleration and environmental factors

To investigate the impact of environmental factors on the aging of epigenetic clocks, we conducted regression analyses adjusting for covariates: age, sex, and 10PCs. We estimated the effects of nine factors on EAAs: BMI, WHR, income level, educational attainment, PWI, drinking status, smoking status, pack-years, and regular exercise. Significant factors were identified using a *p*-value threshold adjusted for Bonferroni correction [*p*-value < 0.05/(nine environmental factors × number of clocks) = 1.11E-03 for first-generation clocks and 1.39E-03 for second-generation clocks].

**FIGURE 3:**
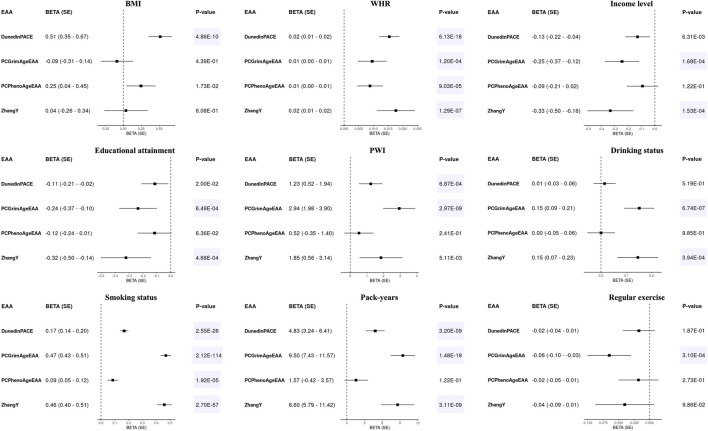
Forest plots for epigenetic age accelerations (EAAs) of second-generation clocks and environmental factors in KARE. Beta values with standard errors (SEs) were displayed along with their corresponding *p*-values. All regression models adjusted for age, sex, and 10 principal components, with all EAAs scaled to a mean of 0 and a standard deviation of 1. Significant results were highlighted with a blue background based on Bonferroni-corrected *p*-values [0.05/(4 × 9) = 1.39E-03].

Most environmental factors did not impact the acceleration of first-generation clocks (Supplementary Table S4). Only smoking had a significant effect on the acceleration of PCHorvath and PCHannum (*p*-value < 1.11E-03). In contrast, EAAs of second-generation clocks were influenced by various environmental factors (Figure 3). Increases in WHR and smoking were associated with accelerated aging across all second-generation clocks (Beta > 0, *p*-value < 1.39E-03). Higher socioeconomic status (income level and educational attainment) slowed down the aging of PCGrimAge and ZhangY, whereas drinking and an increase in pack-years sped up their aging. PCGrimAge was also decelerated by regular exercise and good psychosocial status (PWI), while DunedinPACE increased with higher BMI and an increase in pack-years, as well as poor psychosocial status. As depicted in Figure 3; Supplementary Table S3, when environmental factors significantly influenced the aging of epigenetic clocks, they affected them in the same direction.

### Validation in independent Korean samples

We conducted validation studies on the association of EAAs in an independent Korean cohort, Health Examinees (HEXA) of the KoGES (Kim et al., 2017; Health Examinees Study Group, 2015). The basic characteristics of the 822 HEXA participants were presented in Supplementary Table S5. We derived epigenetic ages using the same eight epigenetic clocks and subsequently calculated EAAs based on them. Descriptive statistics are presented in Supplementary Table S6; Supplementary Figure S3. Females exhibited a significantly decelerated aging rate compared to males across epigenetic clocks (*t*-test *p*-value < 0.05), except Bernabeu (*p*-value = 5.01E-02, Supplementary Table S6).

Consistent with the previous findings (Supplementary Table S3), EAAs from the first-generation epigenetic clocks did not exhibit significant associations with any health outcomes in HEXA (*p*-value > 0.05/(five EAAs × seven health outcomes) = 1.43E-03, Supplementary Table S7). Similarly, associations between EAAs of the second-generation clocks and health outcomes demonstrated consistent effect directions in HEXA (*p*-value < 0.05/(four EAAs × seven health outcomes) = 1.79E-03, Figure 2; Table 3). Accelerated aging in all second-generation clocks was consistently associated with an increased risk of T2D. PCGrimAgeEAA and ZhangY were significantly associated with TG levels, while DunedinPACE showed significant associations with hypertension, AST, ALT, HDL, TG, and hs-CRP. Among the previously significant findings, only the association between PCPhenoAge and AST did not reach the corrected *p*-value threshold in HEXA (*p*-value = 4.37E-03). Despite conducting analyses on time to disease onset using follow-up data, no statistically significant results were identified (Supplementary Figure S4; Supplementary Table S8).

**TABLE 3:** Association results of second-generation epigenetic age acceleration in HEXA.

	Trait	OR (95% CI)/BETA (SE)	*p*-value	Trait	OR (95% CI)/BETA (SE)	*p*-value
DunedinPACE	T2D	1.14 (1.1, 1.17)	1.70E-16	HDL	−2.37 (0.48)	1.08E-06
	Hypertension	1.25 (1.09, 1.44)	1.35E-03	Triglyceride	33.59 (4.63)	9.36E-13
	AST	2.37 (0.71)	9.12E-04	hs-CRP	0.15 (0.05)	1.27E-03
	ALT	3.61 (0.94)	1.21E-04			
PCGrimAgeEAA	T2D	1.15 (1.1, 1.19)	2.90E-11	HDL	−0.57 (0.64)	3.74E-01
	Hypertension	1.1 (0.99, 1.22)	8.56E-02	Triglyceride	32.04 (6.18)	2.70E-07
	AST	2.44 (0.94)	9.39E-03	hs-CRP	0.07 (0.06)	2.32E-01
	ALT	0.91 (1.24)	4.64E-01			
PCPhenoAgeEAA	T2D	1.09 (1.05, 1.13)	2.91E-06	HDL	−1.04 (0.57)	6.94E-02
	Hypertension	1.15 (1.03, 1.29)	1.76E-02	Triglyceride	15.75 (5.57)	4.82E-03
	AST	2.4 (0.84)	4.37E-03	hs-CRP	0.1 (0.05)	6.87E-02
	ALT	3.44 (1.1)	1.90E-03			
ZhangY	T2D	1.21 (1.15, 1.28)	3.49E-12	HDL	1.75 (1.66)	2.93E-01
	Hypertension	1.08 (1, 1.17)	4.54E-02	Triglyceride	41.83 (8.26)	5.02E-07
	AST	2.67 (1.26)	3.35E-02	hs-CRP	0.14 (0.08)	6.91E-02
	ALT	1.75 (1.66)	2.93E-01			
DunedinPACE	BMI	0.03 (0.01)	2.50E-04	PWI	0.008 (0.004)	7.34E-02
	WHR	3.3 (0.53)	8.94E-10	Drink	0.04 (0.05)	4.20E-01
	Income	−0.08 (0.02)	7.23E-05	Smoke	0.56 (0.06)	2.09E-19
	Education	−0.08 (0.02)	4.89E-05	Exercise	−0.33 (0.09)	1.93E-04
PCGrimAgeEAA	BMI	−0.02 (0.01)	6.63E-03	PWI	0.002 (0.003)	5.90E-01
	WHR	1.33 (0.41)	1.32E-03	Drink	0.14 (0.04)	6.21E-04
	Income	−0.07 (0.01)	3.67E-07	Smoke	0.68 (0.04)	6.25E-51
	Education	−0.08 (0.01)	2.84E-08	Exercise	−0.22 (0.07)	1.01E-03
PCPhenoAgeEAA	BMI	0.01 (0.01)	1.33E-01	PWI	−0.002 (0.004)	4.96E-01
	WHR	1.23 (0.46)	8.06E-03	Drink	0.02 (0.05)	7.36E-01
	Income	−0.07 (0.02)	3.85E-05	Smoke	0.18 (0.05)	6.63E-04
	Education	−0.06 (0.02)	3.89E-04	Exercise	−0.25 (0.08)	1.06E-03
ZhangY	BMI	−0.01 (0.01)	2.54E-01	PWI	0.002 (0.002)	3.20E-01
	WHR	1.22 (0.31)	7.82E-05	Drink	0.1 (0.03)	8.29E-04
	Income	−0.06 (0.01)	4.60E-07	Smoke	0.42 (0.03)	1.30E-34
	Education	−0.04 (0.01)	9.56E-05	Exercise	−0.14 (0.05)	5.86E-03

All regression models are adjusted for age, sex, and 10 principal components. All EAAs are scaled to mean = 0 and SD = 1. OR, Odds ratios; CI, confidence interval; SE, standard error; WHR, waist-hip ratio; Income, income level; Education, educational attainment; Exercise, regular exercise.

While EAAs from the first-generation clock showed a significant association solely with smoking status previously (Supplementary Table S4), no significant associations with any environmental factors were observed in HEXA [*p*-value > 0.05/(five EAAs × eight environmental factors) = 1.25E-03, Supplementary Table S9]. In contrast, the associations between EAAs of the second-generation clocks and environmental factors were validated in HEXA (Table 3). Smoking consistently sped up the aging of all second-generation clocks, whereas higher socioeconomic status slowed it down. DunedinPACE increased with higher BMI and WHR but decreased with regular exercise. Higher WHR and drinking accelerated the aging of PCGrimAge and ZhangY, while regular exercise decelerated the aging of PCGrimAge and PCPhenoAge. PWI was the only factor that did not show a validated significant association with EAAs of second-generation clocks.

### Effects of epigenetic age acceleration on health outcomes independent of lifestyle factors

We identified that the EAA of second-generation clocks affects health outcomes and is influenced by environmental factors. Since environmental factors affect both health outcomes and epigenetic age, we performed regression analyses with additional adjustments for lifestyle factors, such as body mass index, waist-hip ratio, smoking status, drinking status, and regular exercise, to evaluate the independent effects of EAAs on health outcomes (Table 4). Even after accounting for these factors, the detrimental effect of higher EAA on health outcomes remained significant in KARE (*p*-value < 0.05). While the statistical significance and effect sizes of EAAs on health outcomes were diminished in most cases, the impact of PCGrimAge on the risk of T2D became slightly stronger (OR 1.14 to 1.16, *p*-value 6.91E-15 to 1.33E-15). In HEXA, the influence of EAAs on health outcomes was maintained (*p*-value < 0.05), except for DunedinPACE on hypertension and AST (*p*-value > 0.05). Similar to KARE, the statistical significance and effect sizes of EAAs were reduced across all cases.

**TABLE 4:** Association results of epigenetic age acceleration adjusted for lifestyle factors.

	Trait	OR (95% CI)/BETA (SE)	*p*-value	OR (95% CI)/BETA (SE)*	*p*-value*
KARE					
DunedinPACE	T2D	1.13 (1.1, 1.16)	1.41E-24	1.1 (1.08, 1.13)	6.99E-16
	Hypertension	1.08 (1.05, 1.11)	7.82E-10	1.06 (1.03, 1.09)	4.71E-06
	AST	1.17 (0.37)	1.34E-03	1.03 (0.38)	7.54E-03
	ALT	2.15 (0.42)	3.67E-07	1.42 (0.43)	1.05E-03
	HDL	−1.72 (0.29)	3.50E-09	−0.92 (0.29)	1.57E-03
	Triglyceride	19.13 (2.67)	1.19E-12	12.91 (2.76)	3.06E-06
	hs-CRP	0.61 (0.11)	2.88E-08	0.59 (0.12)	3.88E-07
	FVC % PRED	−1.91 (0.4)	1.70E-06	−1.71 (0.42)	4.83E-05
	FEV1% PRED	−2.06 (0.48)	1.88E-05	−1.76 (0.51)	5.56E-04
PCGrimAgeEAA	T2D	1.14 (1.1, 1.17)	6.91E-15	1.16 (1.12, 1.20)	1.33E-15
	Triglyceride	17.23 (3.72)	3.78E-06	12.4 (4.18)	3.03E-03
	FEV1% PRED	−2.14 (0.67)	1.38E-03	−1.68 (0.77)	2.93E-02
PCPhenoAgeEAA	T2D	1.09 (1.06, 1.13)	2.97E-09	1.07 (1.04, 1.11)	7.64E-07
	Hypertension	1.06 (1.03, 1.09)	4.18E-04	1.04 (1.01, 1.08)	5.02E-03
	AST	1.52 (0.46)	9.13E-04	1.42 (0.46)	2.12E-03
ZhangY	T2D	1.15 (1.11, 1.21)	1.03E-10	1.14 (1.09, 1.19)	2.05E-08
	Triglyceride	19.65 (5.01)	9.05E-05	11.27 (5.27)	3.25E-02
	FEV1% PRED	−3.73 (0.89)	2.91E-05	−3.15 (0.97)	1.16E-03
HEXA					
DunedinPACE	T2D	1.14 (1.1, 1.17)	1.70E-16	1.1 (1.07, 1.14)	2.87E-08
	Hypertension	1.25 (1.09, 1.44)	1.35E-03	1.04 (1, 1.08)	6.96E-02
	AST	2.37 (0.71)	9.12E-04	1.43 (0.83)	8.36E-02
	ALT	3.61 (0.94)	1.21E-04	2.21 (1.01)	2.92E-02
	HDL	−2.37 (0.48)	1.08E-06	−1.67 (0.52)	1.41E-03
	Triglyceride	33.59 (4.63)	9.36E-13	24.36 (5.26)	4.30E-06
	hs-CRP	0.15 (0.05)	1.27E-03	0.15 (0.05)	6.44E-03
PCGrimAgeEAA	T2D	1.15 (1.1, 1.19)	2.90E-11	1.13 (1.08, 1.19)	1.57E-06
	Triglyceride	32.04 (6.18)	2.70E-07	31.89 (7.7)	3.86E-05
PCPhenoAgeEAA	T2D	1.09 (1.05, 1.13)	2.91E-06	1.06 (1.02, 1.1)	2.48E-03
ZhangY	T2D	1.21 (1.15, 1.28)	3.49E-12	1.18 (1.11, 1.26)	4.06E-07
	Triglyceride	41.83 (8.26)	5.02E-07	35.99 (9.55)	1.77E-04

All regression models are adjusted for age, sex, and 10 principal components. All EAAs are scaled to m ean = 0 and SD = 1. *The regression model was further adjusted for body mass index, waist-hip rati o, smoking status, drinking status, and regular exercise. OR, Odds ratios; CI, confidence interval; SE, standard error.

### Effects of epigenetic clocks developed in East Asian ancestry

To identify the effects of clocks developed in same ancestry, we calculated three epigenetic clocks, PCHorvath, PCHannum, and PCPhenoAge, trained on a Japanese sample using the Transfer Elastic Net method (Tomo and Nakaki, 2024). In both KARE and HEXA, these three clocks showed correlations with the original clocks (Supplementary Figure S5) and demonstrated stronger correlations with chronological age compared to the original clocks (Supplementary Tables S1, S6, S10). Regarding associations with health outcomes, we observed slightly more significant *p*-values, such as the effects of PCPhenoAgeEAA on T2D, hypertension, AST, ALT, and FVC% PRED in the KARE cohort (Figure 2; Supplementary Table S11). Conversely, *p*-values for associations between PCPhenoAgeEAA and most health outcomes were less significant in HEXA (Table 3; Supplementary Table S11). In terms of environmental factors (Supplementary Table S12), a significant association between PCHannumEAA and BMI was observed in both KARE and HEXA, which was not evident with the original clocks. Furthermore, while the association between WHR and PCPhenoAgeEAA became more significant, other associations, such as with smoking status in KARE and with income, education, and regular exercise in HEXA, showed reduced significance. No significant results were observed in the survival analysis, consistent with the findings for the original clock (Supplementary Figure S6; Supplementary Table S13).

## Discussion

Recently, the utilization of epigenetic predictors in healthcare research has experienced remarkable growth. We explored the impact of EAAs on disease risk and health indicators, as well as the influence of various environmental factors on the aging of epigenetic clocks. In the current study, we found that faster aging of second-generation epigenetic clocks was significantly associated with adverse health outcomes, including increased risk of chronic diseases, variations in blood markers levels, and reduced lung functions. Moreover, acceleration of these clocks was associated with the onset of chronic diseases in follow-up data. Unhealthy lifestyles, such as smoking, drinking, high BMI and WHR, and poor psychosocial status, were identified as factors that accelerated the aging of second-generation clocks. Conversely, favorable socioeconomic status and regular exercise were associated with slowed aging. Furthermore, we identified the effects of epigenetic age acceleration on health outcomes, independent of lifestyle factors.

We utilized five first-generation and four second-generation epigenetic clocks. Although these clocks were developed outside of East Asian populations, they exhibited a strong correlation with chronological age and associations with health and lifestyle factors, and, as is well known, they accelerated more in men than in women (Horvath et al., 2016). In contrast, iCAS-DNAmAge, the only epigenetic clock developed using data from East Asian populations, displayed a relatively weak correlation with chronological age and EAA of other clocks (Supplementary Table S1; Figure 1), while accelerating more in women than in men (Supplementary Table S2). Additionally, iCAS-DNAmAge is significantly associated with the time to onset risk of hypertension; however, its acceleration appears to reduce this risk (HR < 1). The three epigenetic clocks trained on an East Asian population showed stronger associations with certain health outcomes compared to the original clocks (Supplementary Tables S11, S12). Our findings suggest that epigenetic clocks developed for different ancestries can be valuable for individuals of East Asian ancestry. Additionally, clocks specifically trained for East Asian populations could enhance predictive performance, and second-generation epigenetic clocks are expected to be more efficient in predicting health outcomes than first-generation clocks.

Epigenetic clocks have only recently been developed, leaving much to be understood from a mechanistic perspective. Biological age is a multifaceted phenomenon, influenced by accumulated cellular damage, environmental exposures, genomic instability, and epigenetic alterations (López-Otín et al., 2023; Zhang et al., 2023). Epigenetic clocks derived from DNA methylation integrate multiple independent mechanisms of age-related change, thus each epigenetic clock reflects different mechanisms (Levine et al., 2022). Moreover, second-generation clocks are trained using a range of health indicators rather than solely age. For instance, PhenoAge captures organismal age and the functional state of many organ systems and tissues (Levine et al., 2018), while DunedinPACE measures the pace of aging (Belsky et al., 2022). PCGrimAge and ZhangY exhibit all-cause mortality, although ZhangY uses CpGs that are not related to aging (Lu et al., 2019; Zhang et al., 2017). These factors can influence our results that each clock exhibits varying performance as a health predictor and is affected differently by diverse environmental factors.

As observed in previous studies (Zhang et al., 2019; Levine et al., 2018; Bell et al., 2019; Faul et al., 2023), second-generation epigenetic clocks exhibited higher accuracy in predicting health outcomes compared to first-generation clocks. Furthermore, accelerated aging of second-generation clocks was associated with increased morbidity rates and shifts in biomarker levels indicating deteriorating health conditions, which in turn correlate with higher mortality rates. For example, T2D and hypertension are common comorbidities and age-related chronic metabolic diseases linked with elevated mortality rates (Petrie et al., 2018; Aune et al., 2021; Rockl et al., 2017). AST and ALT are well-known biomarkers used to indicate liver injury, while TG and hs-CRP are well-established risk factors for cardiovascular disease. Elevated levels of these biomarkers have been shown to increase mortality risk (Chen et al., 2022; Liu et al., 2013; Li et al., 2017). HDL, commonly known as “good cholesterol,” is linked to higher mortality risk at both low and high levels (Zhong et al., 2020). FEV1 and FVC are main results of pulmonary function tests, and it is known that low predicted percentages of these values are associated with higher mortality rates (Magnussen et al., 2017). Therefore, our findings suggest that second-generation clocks could serve as predictors of mortality in East Asian populations.

The environmental factors examined in our study have been reported to influence the aging of epigenetic age. Consistent with our findings, high BMI and WHR accelerate this process (Lin et al., 2021), as does smoking and drinking (Ryan et al., 2020), while regular exercise decelerates it (Quach et al., 2017). Poor socioeconomic and psychosocial status also speeds up epigenetic aging (Schmitz et al., 2022; Palma-Gudiel et al., 2020). In addition, adopting a healthier lifestyle, such as regular exercise (Fitzgerald et al., 2021), smoke cessation (Wu et al., 2019), and losing weight (Yaskolka et al., 2021), is known to reverse epigenetic aging. Likewise, changing other environmental factors associated with EAA to healthier options could not only decelerate epigenetic aging but also potentially make one biologically younger.

Overall, this study underscores the utility of epigenetic clocks in the East Asian population. We evaluated different epigenetic clocks and confirmed that the epigenetic age from second-generation clocks provide valuable health predictions, while also suggesting potential for slowing aging through healthier lifestyles. Although we validated our results using an independent cohort, certain aspects remain unvalidated. In the validation cohort, lung function and pack-years could not be analyzed, and the impact of psychosocial status on EAAs did not reach statistical significance (*p*-value > 0.05). Additionally, the impact of EAAs on disease onset could not be confirmed due to insufficient follow-up cases for T2D (six cases) and failure to meet the statistical threshold in the hypertension analysis. Further investigations with larger sample sizes may clarify the association between them in East Asian populations. Aging is represented by various indicators, and our investigation has shown that each epigenetic clock is linked to different indicators, necessitating a comprehensive interpretation that incorporates multiple epigenetic clocks.

## Methods

### Study population

The present study utilized two independent, community-based genomic cohort datasets from the KoGES (Kim et al., 2017). The first cohort, KARE, consisted of 10,030 individuals aged 40–69 years residing in the urban area of Ansan and the rural area of Ansung. These participants were initially recruited between 2001 and 2002 and underwent biennial follow-up examinations until 2010. For the validation of the results from KARE, we employed the second cohort, HEXA (Health Examinees Study Group, 2015), which comprised 65,642 participants from urban areas. These individuals were recruited between 2004 and 2013 at 38 hospitals and local health-screening centers, following standardized procedures. Epidemiological data for both cohorts were provided by the Korea Centers for Disease Control and Prevention. Written informed consent for participation in the KoGES cohorts was obtained from all study participants and confirmed by the Institutional Review Board.

### Phenotype definition

To classify the participants into T2D case and control groups, we used their responses to the questionnaire on T2D diagnostic history and fasting glucose levels. Participants who answered “Yes” to the questionnaire or had a fasting glucose level above 126 mg/dL were classified as the case group. Conversely, those who answered “No” to the questionnaire and had a fasting glucose level below 126 mg/dL were placed in the control group.

We classified participants into hypertension case and control groups based on their responses to the questionnaire on hypertension diagnosis and their systolic and diastolic blood pressure measurements (SBP and DBP). Participants who answered “Yes” to the questionnaire or had an SBP above 140 mmHg or DBP above 90 mmHg were classified into the case group. Those who answered “No” to the questionnaire and had an SBP below 140 mmHg and DBP below 90 mmHg were placed into the control group. The mean value of two measurements was used for both SBP and DBP.

For the survival analysis, we used follow-up data. Since methylation data from the KARE was generated for the 4th follow-up participants, we examined the data from the 5th to the 9th follow-up surveys. T2D and hypertension cases were classified based on upper criteria. The follow-up time for each participant was determined from the date of the 4th follow-up survey to the date of the follow-up survey at which the participant satisfied the classification criteria. The control groups for T2D and hypertension were defined as participants who met the control criteria in the follow-up data from the 4th to the 9th surveys. The methylation data for HEXA was collected at baseline, and the classification of case and control groups for T2D and hypertension was based on data from the first follow-up survey.

Income level was determined by the average monthly income of the family and divided into eight categories. Education attainment was categorized into nine groups, ranging from not attending school to completing graduate education. PWI comprises 18 items, including 11 positive wellbeing items and 7 negative feeling items such as pain, discomfort, anxiety, and depression. Each question is scored from 0 to 3 for stress level, and the total PWI score is the sum of these subscales. A higher PWI score indicates a higher level of psychosocial stress.

### DNA methylation

Methylation data of KoGES were generated from genomic DNA extracted from the blood of the subjects. Genome-wide DNA methylation was profiled using Illumina Infinium Human Methylation 450k BeadChip which measured methylation approximately 450,000 CpG sites (450K), and Illumina Infinium Human Methylation EPIC BeadChip covers over 850,000 CpG sites (EPIC). Illumina intensity data (IDAT) files were processed using R package minfi (Aryee et al., 2014). We screened for samples where fewer than 95% of probes had a detection *p*-value < 0.01 and applied functional normalization preprocessing (preprocessIllumina function). The beta-value was computed by dividing the intensity of the methylated channel by the sum of the intensities from both the methylated and non-methylated probes. This value reflects the methylation level at each CpG site, ranging between 0 and 1, where higher methylation yields a value closer to 1. For KARE, methylation data from 1,925 subjects were used in the analysis, with 397 from 450K chip and 1,528 from EPIC chip. The data from the 450K and EPIC chips were combined into a single dataset, including only the overlapping CpG sites. Methylation data from 822 HEXA subjects, profiled using EPIC chip, were used in the validation analysis.

Since methylation data of KARE derived from two different chips, we applied the ComBat function from the R package sva to correct for batch effects (Leek et al., 2012). The Supplementary Figure S7 illustrates the pattern of changes in PC after correction.

### Epigenetic clocks and epigenetic age acceleration

We employed a total of eight epigenetic clocks trained on blood samples. Most epigenetic clocks were estimated using R package dnaMethyAge from Github (https://github.com/yiluyucheng/dnaMethyAge) (Wang et al., 2024), with missing CpGs filled using median values from the reference dataset. We calculated four first-generation clocks: PCHorvath (Horvath et al., 2018), PCHannum (Hannum et al., 2013), ZhangQ (Zhang et al., 2019), and Bernabeu (Bernabeu et al., 2023), along with four second-generation clocks: PCPhenoAge (Levine et al., 2018), PCGrimAge (Lu et al., 2019), DunedinPACE (Belsky et al., 2022), and ZhangY (Zhang et al., 2017). We prioritized PC-based clocks whenever feasible to minimize noise and improve accuracy (Higgins-Chen et al., 2022). Additionally, iCAS-DNAmAge was calculated using the beta-values of 65 CpGs from the iCAS-DNAmAge paper (Zheng et al., 2024). For the calculation of epigenetic clocks trained using Transfer Elastic Net on Japanese data (Tomo and Nakaki, 2024), we transformed beta-values of CpGs through PCs according to the workflow implemented on Github (https://github.com/MorganLevineLab/PC-Clocks). We then estimated the three clocks trained on Japanese data (PCHorvath, PCHannum, and PCPhenoAge) using the coefficients available at another GitHub repository (https://github.com/t-yui/TransferENet-EpigeneticClock).

Based on the epigenetic age estimates from the clocks, we computed EAA to assess whether individuals are biologically younger or older than their chronological age. EAAs for the clocks were generated by calculating residuals after regressing epigenetic age on chronological age. Since DunedinPACE and ZhangY directly measure the pace of aging and all-cause mortality, respectively, they can be used as EAAs response variables.

### Statistical analysis

Student’s t-tests, Pearson correlation analyses, and regression analyses were performed using basic packages of R version 4.2.3. To correct for batch effects, we generated 1 to 10 PCs for the methylation data using the prcomp function in R and adjusted for them in the regression analyses. We selected 1 to 10 PCs, identifying the point where the variance explained by the PCs flattened. Elbow curves of the PCs variance were visualized in Supplementary Figure S8. The Kaplan-Meier curves, scatter plots, forest plots, and visualization of the elbow point for PCs were created using the R package ggplot2. Cox regression analysis was conducted using the R package survival, and the correlation matrix was visualized using the R package ggcorrplot.

## Data Availability

Publicly available datasets were analyzed in this study. This data can be found here: https://nih.go.kr/ko/main/contents.do?menuNo&equals;300563.
