# Supplementary material for: Assessing the utility of epigenetic clocks for health prediction in South Korean
Source: Front Aging. 2024 Dec 2;5:1493406. doi: 10.3389/fragi.2024.1493406 (PMC11646986; doi:10.3389/fragi.2024.1493406)
Supplement: Supplementary file 1 [file DataSheet1.docx]

| Table 1. Basic characteristics of 1,925 KARE participants. | | |
| --- | --- | --- |
|  | Males | Females |
| Total N | 1,006 (52.3%) | 919 (47.7%) |
| Chronological age (years)* | 59.6 (8.5) | 60.5 (9) |
| Body mass index* | 24.1 (2.9) | 24.6 (3.2) |
| Waist-hip ratio* | 0.93 (0.06) | 0.92 (0.09) |
| Income level* | 4.5 (2.2) | 3.5 (2.2) |
| Educational attainment* | 3.2 (1.6) | 2.1 (1.4) |
| PWI | 17.2 (8.5) | 18 (8.9) |
| Drinking status* |  |  |
| Never | 235 (23.4%) | 705 (76.8%) |
| Ever | 80 (8%) | 16 (1.7%) |
| Current | 691 (68.7%) | 197 (21.5%) |
| Smoking status* |  |  |
| Never | 255 (25.3%) | 887 (96.6%) |
| Ever | 400 (39.8%) | 13 (1.4%) |
| Current | 351 (34.9%) | 18 (2%) |
| Pack-years of smoking* | 29 (19.9) | 10.6 (10.6) |
| Regular exercise* | 440 (43.7%) | 591 (64.3%) |
| Type 2 diabetes* | 332 (33%) | 248 (27%) |
| Hypertension | 436 (43.3%) | 411 (44.7%) |
| AST* (IU/L) | 28.5 (16.1) | 24.1 (10) |
| ALT* (IU/L) | 28.2 (18.3) | 21.4 (12.6) |
| HDL* (mg/dL) | 42 (11.2) | 44.4 (10.8) |
| Triglyceride* (mg/dL) | 143.2 (77.4) | 132.5 (70.6) |
| hs-CRP* (mg/L) | 1.9 (5.2) | 1.4 (2.7) |
| FVC % PRED* | 101.3 (13.1) | 107.9 (14.9) |
| FEV1% PRED* | 106.4 (15.8) | 118.2 (18) |
| All data are presented as mean ± standard deviation or numbers (%). Missing samples for each value are excluded. * P-value of t-test between male and female is less than 0.05. PWI, psychosocial well-being index; AST, alanine aminotransferase; ALT, aspartate aminotransferase; HDL, high density lipoprotein; hs-CRP, high sensitivity C-reactive protein; FVC % PRED, percentage of predicted FVC; FEV1% PRED, percentage of predicted FEV1. | | |

| Table 2. Cox regression analysis of epigenetic age acceleration for onset of type 2 diabetes and hypertension. | | | | |
| --- | --- | --- | --- | --- |
|  |  | Disease | HR (95% CI) | P-value |
| First-generation | BernabeuEAA | T2D | 0.81 (0.66, 0.98) | 3.23E-02 |
|  |  | Hypertension | 0.89 (0.78, 1.01) | 6.52E-02 |
|  | iCAS-DNAmAgeEAA | T2D | 0.77 (0.59, 1.01) | 6.03E-02 |
|  |  | Hypertension | 0.78 (0.66, 0.92) | 3.32E-03 |
|  | PCHannumEAA | T2D | 0.75 (0.56, 1.02) | 6.87E-02 |
|  |  | Hypertension | 0.90 (0.74, 1.1) | 3.09E-01 |
|  | PCHorvathEAA | T2D | 0.96 (0.73, 1.27) | 7.84E-01 |
|  |  | Hypertension | 0.84 (0.7, 1.01) | 6.60E-02 |
|  | ZhangQEAA | T2D | 0.87 (0.71, 1.07) | 1.84E-01 |
|  |  | Hypertension | 0.97 (0.84, 1.11) | 6.65E-01 |
| Second-generation | DunedinPACE | T2D | 1.99 (1.60, 2.48) | 7.00E-10 |
|  |  | Hypertension | 1.28 (1.12, 1.47) | 3.66E-04 |
|  | PCGrimAgeEAA | T2D | 1.50 (1.14, 1.98) | 3.65E-03 |
|  |  | Hypertension | 1.06 (0.87, 1.3) | 5.36E-01 |
|  | PCPhenoAgeEAA | T2D | 1.05 (0.81, 1.37) | 7.10E-01 |
|  |  | Hypertension | 1.02 (0.86, 1.22) | 8.11E-01 |
|  | ZhangY | T2D | 2.34 (1.60, 3.41) | 1.06E-05 |
|  |  | Hypertension | 1.55 (1.20, 2) | 7.18E-04 |
| All Cox models are adjusted for age, sex, and 10 principal components. All EAAs are scaled to mean = 0 and SD = 1. HR, Hazard ratios; CI, confidence interval. | | | | |

| Table 3. Association results of second-generation epigenetic age acceleration in HEXA. | | | | | | |
| --- | --- | --- | --- | --- | --- | --- |
|  | Trait | OR (95% CI) /  BETA (SE) | P-value | Trait | OR (95% CI) / BETA (SE) | P-value |
| DunedinPACE | T2D | 1.14 (1.1, 1.17) | 1.70E-16 | HDL | -2.37 (0.48) | 1.08E-06 |
|  | Hypertension | 1.25 (1.09, 1.44) | 1.35E-03 | Triglyceride | 33.59 (4.63) | 9.36E-13 |
|  | AST | 2.37 (0.71) | 9.12E-04 | hs-CRP | 0.15 (0.05) | 1.27E-03 |
|  | ALT | 3.61 (0.94) | 1.21E-04 |  |  |  |
| PCGrimAgeEAA | T2D | 1.15 (1.1, 1.19) | 2.90E-11 | HDL | -0.57 (0.64) | 3.74E-01 |
|  | Hypertension | 1.1 (0.99, 1.22) | 8.56E-02 | Triglyceride | 32.04 (6.18) | 2.70E-07 |
|  | AST | 2.44 (0.94) | 9.39E-03 | hs-CRP | 0.07 (0.06) | 2.32E-01 |
|  | ALT | 0.91 (1.24) | 4.64E-01 |  |  |  |
| PCPhenoAgeEAA | T2D | 1.09 (1.05, 1.13) | 2.91E-06 | HDL | -1.04 (0.57) | 6.94E-02 |
|  | Hypertension | 1.15 (1.03, 1.29) | 1.76E-02 | Triglyceride | 15.75 (5.57) | 4.82E-03 |
|  | AST | 2.4 (0.84) | 4.37E-03 | hs-CRP | 0.1 (0.05) | 6.87E-02 |
|  | ALT | 3.44 (1.1) | 1.90E-03 |  |  |  |
| ZhangY | T2D | 1.21 (1.15, 1.28) | 3.49E-12 | HDL | 1.75 (1.66) | 2.93E-01 |
|  | Hypertension | 1.08 (1, 1.17) | 4.54E-02 | Triglyceride | 41.83 (8.26) | 5.02E-07 |
|  | AST | 2.67 (1.26) | 3.35E-02 | hs-CRP | 0.14 (0.08) | 6.91E-02 |
|  | ALT | 1.75 (1.66) | 2.93E-01 |  |  |  |
| DunedinPACE | BMI | 0.03 (0.01) | 2.50E-04 | PWI | 0.008 (0.004) | 7.34E-02 |
|  | WHR | 3.3 (0.53) | 8.94E-10 | Drink | 0.04 (0.05) | 4.20E-01 |
|  | Income | -0.08 (0.02) | 7.23E-05 | Smoke | 0.56 (0.06) | 2.09E-19 |
|  | Education | -0.08 (0.02) | 4.89E-05 | Exercise | -0.33 (0.09) | 1.93E-04 |
| PCGrimAgeEAA | BMI | -0.02 (0.01) | 6.63E-03 | PWI | 0.002 (0.003) | 5.90E-01 |
|  | WHR | 1.33 (0.41) | 1.32E-03 | Drink | 0.14 (0.04) | 6.21E-04 |
|  | Income | -0.07 (0.01) | 3.67E-07 | Smoke | 0.68 (0.04) | 6.25E-51 |
|  | Education | -0.08 (0.01) | 2.84E-08 | Exercise | -0.22 (0.07) | 1.01E-03 |
| PCPhenoAgeEAA | BMI | 0.01 (0.01) | 1.33E-01 | PWI | -0.002 (0.004) | 4.96E-01 |
|  | WHR | 1.23 (0.46) | 8.06E-03 | Drink | 0.02 (0.05) | 7.36E-01 |
|  | Income | -0.07 (0.02) | 3.85E-05 | Smoke | 0.18 (0.05) | 6.63E-04 |
|  | Education | -0.06 (0.02) | 3.89E-04 | Exercise | -0.25 (0.08) | 1.06E-03 |
| ZhangY | BMI | -0.01 (0.01) | 2.54E-01 | PWI | 0.002 (0.002) | 3.20E-01 |
|  | WHR | 1.22 (0.31) | 7.82E-05 | Drink | 0.1 (0.03) | 8.29E-04 |
|  | Income | -0.06 (0.01) | 4.60E-07 | Smoke | 0.42 (0.03) | 1.30E-34 |
|  | Education | -0.04 (0.01) | 9.56E-05 | Exercise | -0.14 (0.05) | 5.86E-03 |
| All regression models are adjusted for age, sex, and 10 principal components. All EAAs are scaled to mean = 0 and SD = 1. OR, Odds ratios; CI, confidence interval; SE, standard error; WHR, waist-hip ratio; Income, income level; Education, educational attainment; Exercise, regular exercise. | | | | | | |

| Table 4. Association results of epigenetic age acceleration adjusted for lifestyle factors. | | | | | | |
| --- | --- | --- | --- | --- | --- | --- |
|  | Trait | OR (95% CI) /  BETA (SE) | P-value |  | OR (95% CI) /  BETA (SE)* | P-value* |
| KARE |  |  |  |  |  |  |
| DunedinPACE | T2D | 1.13 (1.1, 1.16) | 1.41E-24 |  | 1.1 (1.08, 1.13) | 6.99E-16 |
|  | Hypertension | 1.08 (1.05, 1.11) | 7.82E-10 |  | 1.06 (1.03, 1.09) | 4.71E-06 |
|  | AST | 1.17 (0.37) | 1.34E-03 |  | 1.03 (0.38) | 7.54E-03 |
|  | ALT | 2.15 (0.42) | 3.67E-07 |  | 1.42 (0.43) | 1.05E-03 |
|  | HDL | -1.72 (0.29) | 3.50E-09 |  | -0.92 (0.29) | 1.57E-03 |
|  | Triglyceride | 19.13 (2.67) | 1.19E-12 |  | 12.91 (2.76) | 3.06E-06 |
|  | hs-CRP | 0.61 (0.11) | 2.88E-08 |  | 0.59 (0.12) | 3.88E-07 |
|  | FVC % PRED | -1.91 (0.4) | 1.70E-06 |  | -1.71 (0.42) | 4.83E-05 |
|  | FEV1% PRED | -2.06 (0.48) | 1.88E-05 |  | -1.76 (0.51) | 5.56E-04 |
| PCGrimAgeEAA | T2D | 1.14 (1.1, 1.17) | 6.91E-15 |  | 1.16 (1.12, 1.20) | 1.33E-15 |
|  | Triglyceride | 17.23 (3.72) | 3.78E-06 |  | 12.4 (4.18) | 3.03E-03 |
|  | FEV1% PRED | -2.14 (0.67) | 1.38E-03 |  | -1.68 (0.77) | 2.93E-02 |
| PCPhenoAgeEAA | T2D | 1.09 (1.06, 1.13) | 2.97E-09 |  | 1.07 (1.04, 1.11) | 7.64E-07 |
|  | Hypertension | 1.06 (1.03, 1.09) | 4.18E-04 |  | 1.04 (1.01, 1.08) | 5.02E-03 |
|  | AST | 1.52 (0.46) | 9.13E-04 |  | 1.42 (0.46) | 2.12E-03 |
| ZhangY | T2D | 1.15 (1.11, 1.21) | 1.03E-10 |  | 1.14 (1.09, 1.19) | 2.05E-08 |
|  | Triglyceride | 19.65 (5.01) | 9.05E-05 |  | 11.27 (5.27) | 3.25E-02 |
|  | FEV1% PRED | -3.73 (0.89) | 2.91E-05 |  | -3.15 (0.97) | 1.16E-03 |
| HEXA |  |  |  |  |  |  |
| DunedinPACE | T2D | 1.14 (1.1, 1.17) | 1.70E-16 |  | 1.1 (1.07, 1.14) | 2.87E-08 |
|  | Hypertension | 1.25 (1.09, 1.44) | 1.35E-03 |  | 1.04 (1, 1.08) | 6.96E-02 |
|  | AST | 2.37 (0.71) | 9.12E-04 |  | 1.43 (0.83) | 8.36E-02 |
|  | ALT | 3.61 (0.94) | 1.21E-04 |  | 2.21 (1.01) | 2.92E-02 |
|  | HDL | -2.37 (0.48) | 1.08E-06 |  | -1.67 (0.52) | 1.41E-03 |
|  | Triglyceride | 33.59 (4.63) | 9.36E-13 |  | 24.36 (5.26) | 4.30E-06 |
|  | hs-CRP | 0.15 (0.05) | 1.27E-03 |  | 0.15 (0.05) | 6.44E-03 |
| PCGrimAgeEAA | T2D | 1.15 (1.1, 1.19) | 2.90E-11 |  | 1.13 (1.08, 1.19) | 1.57E-06 |
|  | Triglyceride | 32.04 (6.18) | 2.70E-07 |  | 31.89 (7.7) | 3.86E-05 |
| PCPhenoAgeEAA | T2D | 1.09 (1.05, 1.13) | 2.91E-06 |  | 1.06 (1.02, 1.1) | 2.48E-03 |
| ZhangY | T2D | 1.21 (1.15, 1.28) | 3.49E-12 |  | 1.18 (1.11, 1.26) | 4.06E-07 |
|  | Triglyceride | 41.83 (8.26) | 5.02E-07 |  | 35.99 (9.55) | 1.77E-04 |
| All regression models are adjusted for age, sex, and 10 principal components. All EAAs are scaled to mean = 0 and SD = 1. * The regression model was further adjusted for body mass index, waist-hip ratio, smoking status, drinking status, and regular exercise. OR, Odds ratios; CI, confidence interval; SE, standard error; | | | | | | |

| Table S1. Descriptive statistics for epigenetic clocks of 1,925 KARE participants. | | | | |
| --- | --- | --- | --- | --- |
|  |  | Mean (SD) | Correlation coefficient with cAge* | Mean (SD) of EAA |
| First-generation | Bernabeu | 60.98 (8.51) | 0.96 | 0.0 (2.47) |
|  | iCAS-DNAmAge | 2.52 (0.25) | 0.79 | 0.0 (0.15) |
|  | ZhangQ | 58.6 (7.9) | 0.94 | 0.0 (2.65) |
|  | PCHannum | 65.32 (7.25) | 0.82 | 0.0 (4.11) |
|  | PCHorvath | 55.54 (6.46) | 0.77 | 0.0 (4.13) |
| Second-generation | PCGrimAge | 69.1 (7.65) | 0.9 | 0.0 (3.32) |
|  | PCPhenoAge | 57.95 (8.45) | 0.83 | 0.0 (4.71) |
|  | DunedinPACE | 1.05 (0.11) | 0.36 |  |
|  | ZhangY | -2.61 (0.36) | 0.27 |  |
| * Pearson correlation coefficient. EAA, epigenetic age acceleration; cAge, chronological age; | | | | |

| Table S2. Statistics for epigenetic age acceleration measures of KARE participants. | | | | |
| --- | --- | --- | --- | --- |
|  |  | Males | Females | P-value* |
| First-generation | BernabeuEAA | 0.13 (2.5) | -0.14 (2.43) | 1.60E-02 |
|  | iCAS-DNAmAgeEAA | -0.007 (0.16) | 0.008 (0.14) | 2.72E-02 |
|  | ZhangQEAA | 0.39 (2.63) | -0.43 (2.6) | 6.39E-12 |
|  | PCHannumEAA | 0.9 (4.13) | -0.98 (3.85) | 1.64E-24 |
|  | PCHorvathEAA | 0.56 (4.04) | -0.61 (4.14) | 4.36E-10 |
| Second-generation | PCGrimAgeEAA | 2.12 (2.79) | -2.32 (2.06) | 6.35E-253 |
|  | PCPhenoAgeEAA | 0.87 (4.45) | -0.96 (4.8) | 1.12E-17 |
|  | DunedinPACE | 1.08 (0.11) | 1.02 (0.1) | 6.86E-31 |
|  | ZhangY | -2.48 (0.34) | -2.75 (0.32) | 3.22E-69 |
| All data are presented as mean ± standard deviation. * P-value of t-test between male and female. | | | | |

| Table S3. Association between first-generation epigenetic age acceleration and health outcomes in KARE. | | | | | | |
| --- | --- | --- | --- | --- | --- | --- |
|  | Trait | OR (95% CI) /  BETA (SE) | P-value | Trait | OR (95% CI) /  BETA (SE) | P-value |
| BernabeuEAA | T2D | 1.01 (0.99, 1.03) | 3.24E-01 | Triglyceride | -1.269 (2.465) | 6.07E-01 |
|  | Hypertension | 1.03 (1.01, 1.05) | 1.09E-02 | hs-CRP | -0.078 (0.101) | 4.39E-01 |
|  | AST | 0.559 (0.333) | 9.32E-02 | FVC % PRED | 0.002 (0.367) | 9.96E-01 |
|  | ALT | 0.708 (0.386) | 6.71E-02 | FEV1% PRED | -0.013 (0.443) | 9.76E-01 |
|  | HDL | 0.007 (0.265) | 9.80E-01 |  |  |  |
| iCAS-DNAmAgeEAA | T2D | 1.03 (1, 1.06) | 6.03E-02 | Triglyceride | -4.409 (3.210) | 1.70E-01 |
|  | Hypertension | 1.01 (0.98, 1.04) | 7.25E-01 | hs-CRP | 0.134 (0.132) | 3.07E-01 |
|  | AST | 1.116 (0.433) | 1.01E-02 | FVC % PRED | 0.016 (0.478) | 9.73E-01 |
|  | ALT | 0.796 (0.503) | 1.14E-01 | FEV1% PRED | 0.245 (0.577) | 6.71E-01 |
|  | HDL | -0.461 (0.346) | 1.83E-01 |  |  |  |
| ZhangQEAA | T2D | 1.03 (1.01, 1.05) | 1.33E-02 | Triglyceride | -1.204 (2.561) | 6.38E-01 |
|  | Hypertension | 1.03 (1.01, 1.06) | 8.07E-03 | hs-CRP | -0.026 (0.105) | 8.07E-01 |
|  | AST | 0.529 (0.346) | 1.26E-01 | FVC % PRED | 0.059 (0.385) | 8.77E-01 |
|  | ALT | 0.443 (0.402) | 2.70E-01 | FEV1% PRED | 0.318 (0.465) | 4.93E-01 |
|  | HDL | -0.076 (0.276) | 7.83E-01 |  |  |  |
| PCHannumEAA | T2D | 1.02 (0.99, 1.06) | 1.55E-01 | Triglyceride | 0.843 (3.723) | 8.21E-01 |
|  | Hypertension | 1.04 (1.01, 1.08) | 2.43E-02 | hs-CRP | 0.04 (0.153) | 7.91E-01 |
|  | AST | 0.849 (0.503) | 9.15E-02 | FVC % PRED | -0.523 (0.557) | 3.48E-01 |
|  | ALT | 0.520 (0.584) | 3.73E-01 | FEV1% PRED | -0.050 (0.672) | 9.41E-01 |
|  | HDL | 0.134 (0.401) | 7.39E-01 |  |  |  |
| PCHorvathEAA | T2D | 1.02 (0.99, 1.05) | 1.25E-01 | Triglyceride | 3.689 (3.428) | 2.82E-01 |
|  | Hypertension | 1.03 (1, 1.07) | 4.36E-02 | hs-CRP | 0.021 (0.14) | 8.84E-01 |
|  | AST | 0.243 (0.463) | 6.00E-01 | FVC % PRED | -0.369 (0.513) | 4.71E-01 |
|  | ALT | 0.034 (0.538) | 9.50E-01 | FEV1% PRED | 0.027 (0.619) | 9.66E-01 |
|  | HDL | -0.199 (0.369) | 5.90E-01 |  |  |  |
| All regression models are adjusted for age, sex, and 10 principal components. All EAAs are scaled to mean = 0 and SD = 1. OR, Odds ratios; CI, confidence interval; SE, standard error. | | | | | | |

| Table S4. Association between first-generation epigenetic age acceleration and environmental factors in KARE. | | | | | | |
| --- | --- | --- | --- | --- | --- | --- |
|  | Trait | BETA (SE) | P-value | Trait | BETA (SE) | P-value |
| BernabeuEAA | BMI | 0.007 (0.007) | 2.94E-01 | Drink | -0.03 (0.026) | 2.50E-01 |
|  | WHR | -0.718 (0.319) | 2.46E-02 | Smoke | -0.059 (0.036) | 1.01E-01 |
|  | Income | -0.001 (0.013) | 9.62E-01 | Pack-years | -0.002 (0.002) | 1.86E-01 |
|  | Education | -0.005 (0.021) | 8.18E-01 | Exercise | 0.021 (0.044) | 6.30E-01 |
|  | PWI | -0.001 (0.004) | 8.31E-01 |  |  |  |
| iCAS-DNAmAgeEAA | BMI | 0.000 (0.005) | 9.37E-01 | Drink | -0.01 (0.020) | 6.06E-01 |
|  | WHR | 0.061 (0.245) | 8.05E-01 | Smoke | 0.046 (0.028) | 9.27E-02 |
|  | Income | -0.009 (0.010) | 3.61E-01 | Pack-years | -0.001 (0.001) | 3.06E-01 |
|  | Education | -0.017 (0.016) | 3.09E-01 | Exercise | -0.029 (0.034) | 3.87E-01 |
|  | PWI | 0.001 (0.003) | 7.61E-01 |  |  |  |
| PCHannumEAA | BMI | 0.009 (0.005) | 6.14E-02 | Drink | 0.01 (0.017) | 5.70E-01 |
|  | WHR | 0.078 (0.212) | 7.12E-01 | Smoke | 0.082 (0.024) | 5.21E-04 |
|  | Income | 0.004 (0.008) | 6.58E-01 | Pack-years | 0.001 (0.001) | 2.22E-01 |
|  | Education | -0.002 (0.014) | 8.97E-01 | Exercise | -0.032 (0.029) | 2.67E-01 |
|  | PWI | 0.003 (0.002) | 2.73E-01 |  |  |  |
| PCHorvathEAA | BMI | 0.007 (0.005) | 1.56E-01 | Drink | -0.012 (0.019) | 5.36E-01 |
|  | WHR | 0.234 (0.229) | 3.08E-01 | Smoke | 0.105 (0.026) | 4.74E-05 |
|  | Income | -0.002 (0.009) | 8.13E-01 | Pack-years | 0.003 (0.001) | 3.45E-02 |
|  | Education | 0.001 (0.015) | 9.24E-01 | Exercise | -0.038 (0.032) | 2.26E-01 |
|  | PWI | 0.004 (0.003) | 1.49E-01 |  |  |  |
| ZhangQEAA | BMI | 0.009 (0.007) | 1.66E-01 | Drink | -0.009 (0.025) | 7.07E-01 |
|  | WHR | -0.133 (0.308) | 6.66E-01 | Smoke | 0.064 (0.035) | 6.22E-02 |
|  | Income | 0.000 (0.012) | 9.82E-01 | Pack-years | -0.001 (0.002) | 6.68E-01 |
|  | Education | -0.014 (0.020) | 4.84E-01 | Exercise | 0.021 (0.043) | 6.26E-01 |
|  | PWI | 0.005 (0.004) | 1.98E-01 |  |  |  |
| All regression models are adjusted for age, sex, and 10 principal components. All EAAs are scaled to mean = 0 and SD = 1. SE, standard error; WHR, waist-hip ratio; Income, income level; Education, educational attainment; Exercise, regular exercise. | | | | | | |

| Table S5. Basic characteristics of 822 HEXA participants. | | |
| --- | --- | --- |
|  | Males | Females |
| Total N | 622 (75.7%) | 200 (24.3%)) |
| Chronological age (years)* | 49.5 (6.1) | 54.1 (3) |
| Body mass index* | 25.2 (4.6) | 23.6 (2.2) |
| Waist-hip ratio* | 0.9 (0.06) | 0.85 (0.06) |
| Income level* | 5.66 (1.6) | 5.3 (1.8) |
| Educational attainment* | 5.1 (1.8) | 4 (1.6) |
| PWI | 15.3 (7.5) | 16 (7.3) |
| Drinking status* |  |  |
| Never | 33 (5.3%) | 145 (72.5%) |
| Ever | 32 (5.2%) | 5 (2.5%) |
| Current | 557 (89.5%) | 50 (25%) |
| Smoking status* |  |  |
| Never | 46 (8.9%) | 192 (96%) |
| Ever | 196 (37.8%) | 5 (2.5%) |
| Current | 277 (53.4%) | 3 (1.5%) |
| Regular exercise* | 1.2 (0.3) | 1.5 (0.5) |
| Type 2 diabetes* | 146 (23.5%) | 100 (50%) |
| Hypertension* | 195 (31.4%) | 45 (22.5%) |
| AST* (IU/L) | 29.9 (19.2) | 25.3 (15.9) |
| ALT* (IU/L) | 33.8 (25) | 26.1 (24.7) |
| HDL* (mg/dL) | 49.4 (12.1) | 54.7 (13.4) |
| Triglyceride* (mg/dL) | 160.2 (93.2) | 133.8 (87.2) |
| hs-CRP (mg/L) | 0.2 (0.6) | 0.4 (2.0) |
| All data are presented as mean ± standard deviation or numbers (%). Missing samples for each value are excluded. * p-value of t-test between male and female is less than 0.05. PWI, psychosocial well-being index; AST, alanine aminotransferase; ALT, aspartate aminotransferase; HDL, high density lipoprotein; hs-CRP, high sensitivity C-reactive protein; | | |

| Table S6. Descriptive statistics for epigenetic clocks of HEXA participants. | | | | |
| --- | --- | --- | --- | --- |
|  |  | Male ^a^ | Female ^a^ | Correlation coefficient ^b^ |
| First-generation | Bernabeu | 51.94(6.70) | 56.11(3.47) | 0.93 |
|  | BernabeuEAA | 0.09(2.38) | -0.29(2.42) | 0 |
|  | iCAS-DNAmAge | -0.53 (0.29) | -0.51 (0.21) | 0.7 |
|  | iCAS-DNAmAgeEAA* | 0.03 (0.19) | -0.1 (0.18) | 0 |
|  | ZhangQ | 48.27(6.60) | 51.57(3.58) | 0.91 |
|  | ZhangQEAA* | 0.26(2.55) | -0.80(2.52) | 0 |
|  | PCHannum | 61.93(5.44) | 60.55(3.65) | 0.56 |
|  | PCHannumEAA* | 0.87(4.19) | -2.71(3.09) | 0 |
|  | PCHorvath | 52.45(5.38) | 51.33(3.75) | 0.46 |
|  | PCHorvathEAA* | 0.71(4.60) | -2.20(3.32) | 0 |
| Second-generation | PCGrimAge | 63.86(5.21) | 62.39(3.02) | 0.73 |
|  | PCGrimAgeEAA* | 1.02(2.99) | -3.16(2.03) | 0 |
|  | PCPhenoAge | 52.46(6.65) | 54.89(4.36) | 0.73 |
|  | PCPhenoAgeEAA* | 0.28(4.37) | -0.86(3.70) | 0 |
|  | DunedinPACE* | 1.05(0.11) | 0.99(0.10) | 0.08 |
|  | ZhangY* | -2.63(0.33) | -2.83(0.29) | -0.03 |
| * P-value of t-test between male and female < 0.05. a. Data are presented as mean ± standard deviation; b. Pearson correlation coefficient with chronological age; EAA, epigenetic age acceleration; | | | | |

| Table S7. Association between first-generation epigenetic age acceleration and health outcomes in HEXA. | | | | | | |
| --- | --- | --- | --- | --- | --- | --- |
|  | Trait | OR (95% CI) /  BETA (SE) | P-value | Trait | OR (95% CI) / BETA (SE) | P-value |
| BernabeuEAA | T2D | 0.98 (0.95, 1.01) | 1.42E-01 | HDL | 0.335 (0.462) | 4.68E-01 |
|  | Hypertension | 1.02 (0.98, 1.05) | 3.08E-01 | Triglyceride | 3.998 (4.497) | 3.74E-01 |
|  | AST | 0.819 (0.676) | 2.26E-01 | hs-CRP | -0.032 (0.043) | 4.61E-01 |
|  | ALT | 1.013 (0.890) | 2.55E-01 |  |  |  |
| iCAS-DNAmAgeEAA | T2D | 0.99 (0.96, 1.03) | 6.93E-01 | HDL | 0.636 (0.498) | 2.02E-01 |
|  | Hypertension | 1 (0.97, 1.04) | 1.00E+00 | Triglyceride | 1.420 (4.860) | 7.70E-01 |
|  | AST | 1.595 (0.728) | 2.88E-02 | hs-CRP | 0.044 (0.047) | 3.49E-01 |
|  | ALT | 1.286 (0.961) | 1.81E-01 |  |  |  |
| PCHannumEAA | T2D | 1.02 (0.97, 1.06) | 4.92E-01 | HDL | -0.950 (0.691) | 1.70E-01 |
|  | Hypertension | 1.05 (1, 1.1) | 7.52E-02 | Triglyceride | 7.858 (6.752) | 2.45E-01 |
|  | AST | 0.389 (1.014) | 7.02E-01 | hs-CRP | -0.110 (0.064) | 8.67E-02 |
|  | ALT | 1.168 (1.334) | 3.82E-01 |  |  |  |
| PCHorvathEAA | T2D | 1.01 (0.97, 1.05) | 5.79E-01 | HDL | -1.431 (0.643) | 2.62E-02 |
|  | Hypertension | 1.02 (0.97, 1.07) | 4.02E-01 | Triglyceride | 11.431 (6.277) | 6.90E-02 |
|  | AST | -1.735 (0.943) | 6.62E-02 | hs-CRP | -0.101 (0.060) | 9.32E-02 |
|  | ALT | -0.908 (1.244) | 4.66E-01 |  |  |  |
| ZhangQEAA | T2D | 1 (0.97, 1.03) | 8.48E-01 | HDL | 0.321 (0.477) | 5.01E-01 |
|  | Hypertension | 1.01 (0.98, 1.05) | 5.08E-01 | Triglyceride | 4.468 (4.649) | 3.37E-01 |
|  | AST | 0.499 (0.697) | 4.74E-01 | hs-CRP | -0.006 (0.045) | 8.87E-01 |
|  | ALT | 0.654 (0.918) | 4.76E-01 |  |  |  |
| All regression models are adjusted for age, sex, and 10 principal components. All EAAs are scaled to mean = 0 and SD = 1. OR, Odds ratios; CI, confidence interval; SE, standard error. | | | | | | |

| Table S8. Cox regression analysis of epigenetic age acceleration for onset of hypertension in HEXA. | | | |
| --- | --- | --- | --- |
|  |  | HR (95% CI) | P-value |
| First-generation | BernabeuEAA | 0.78 (0.55, 1.10) | 1.53E-01 |
|  | iCAS-DNAmAgeEAA | 0.87 (0.60, 1.23) | 4.52E-01 |
|  | PCHannumEAA | 0.52 (0.25, 1.06) | 7.02E-02 |
|  | PCHorvathEAA | 0.63 (0.35, 1.14) | 1.29E-01 |
|  | ZhangQEAA | 0.70 (0.47, 1.03) | 6.78E-02 |
| Second-generation | DunedinPACE | 1.29 (0.88, 1.88) | 1.87E-01 |
|  | PCGrimAgeEAA | 1.24 (0.75, 2.04) | 4.07E-01 |
|  | PCPhenoAgeEAA | 0.61 (0.38, 0.99) | 4.51E-02 |
|  | ZhangY | 0.98 (0.48, 2.00) | 9.59E-01 |
| All Cox models are adjusted for age, sex, and 10 principal components. All EAAs are scaled to mean = 0 and SD = 1. HR, Hazard ratios; CI, confidence interval. | | | |

| Table S9. Association between first-generation epigenetic age acceleration and environmental factors in HEXA. | | | | | | |
| --- | --- | --- | --- | --- | --- | --- |
|  | Trait | BETA (SE) | P-value | Trait | BETA (SE) | P-value |
| BernabeuEAA | BMI | 0.004 (0.009) | 6.55E-01 | PWI | -0.009 (0.004) | 5.25E-02 |
|  | WHR | 0.086 (0.578) | 8.81E-01 | Drink | -0.050 (0.056) | 3.74E-01 |
|  | Income | 0.012 (0.021) | 5.72E-01 | Smoke | -0.083 (0.067) | 2.13E-01 |
|  | Education | 0.003 (0.020) | 8.63E-01 | Exercise | -0.085 (0.093) | 3.65E-01 |
| iCAS-DNAmAgeEAA | BMI | -0.00 (0.009) | 9.88E-01 | PWI | -0.007 (0.004) | 7.28E-02 |
|  | WHR | 0.620 (0.535) | 2.47E-01 | Drink | 0.061 (0.052) | 2.40E-01 |
|  | Income | -0.028 (0.019) | 1.54E-01 | Smoke | 0.043 (0.061) | 4.79E-01 |
|  | Education | -0.013 (0.018) | 4.63E-01 | Exercise | -0.131 (0.087) | 1.30E-01 |
| PCHannumEAA | BMI | 0.018 (0.006) | 3.89E-03 | PWI | -0.007 (0.003) | 2.88E-02 |
|  | WHR | 0.837 (0.385) | 2.99E-02 | Drink | 0.024 (0.037) | 5.12E-01 |
|  | Income | -0.024 (0.013) | 5.88E-02 | Smoke | 0.079 (0.044) | 7.49E-02 |
|  | Education | -0.035 (0.013) | 7.42E-03 | Exercise | -0.165 (0.062) | 8.17E-03 |
| PCHorvathEAA | BMI | 0.007 (0.007) | 2.61E-01 | PWI | -0.006 (0.003) | 5.07E-02 |
|  | WHR | 0.402 (0.414) | 3.32E-01 | Drink | 0.038 (0.040) | 3.44E-01 |
|  | Income | -0.022 (0.014) | 1.22E-01 | Smoke | 0.086 (0.048) | 7.07E-02 |
|  | Education | -0.034 (0.014) | 1.61E-02 | Exercise | -0.168 (0.067) | 1.18E-02 |
| ZhangQEAA | BMI | 0.001 (0.009) | 8.85E-01 | PWI | -0.011 (0.004) | 1.15E-02 |
|  | WHR | 0.523 (0.560) | 3.50E-01 | Drink | -0.016 (0.054) | 7.63E-01 |
|  | Income | -0.021 (0.020) | 3.08E-01 | Smoke | 0.116 (0.065) | 7.26E-02 |
|  | Education | -0.005 (0.019) | 8.03E-01 | Exercise | -0.122 (0.090) | 1.79E-01 |
| All regression models are adjusted for age, sex, and 10 principal components. All EAAs are scaled to mean = 0 and SD = 1. SE, standard error; WHR, waist-hip ratio; Income, income level; Education, educational attainment; Exercise, regular exercise. | | | | | | |

| Table S10. Descriptive statistics for epigenetic clocks trained using Transfer Elastic Net on Japanese data. | | | | |
| --- | --- | --- | --- | --- |
|  |  | Male ^a^ | Female ^a^ | Correlation coefficient ^b^ |
| KARE | PCHorvath | 1.57 (0.38) | 1.55 (0.37) | 0.85 |
|  | PCHorvathEAA* | 0.02 (0.2) | -0.02 (0.19) | 0 |
|  | PCHannum | 55.05 (8.78) | 55.37 (8.56) | 0.86 |
|  | PCHannumEAA* | 0.2 (4.37) | -0.22 (4.37) | 0 |
|  | PCPhenoAge | 51.83 (9.18) | 51.18 (8.89) | 0.84 |
|  | PCPhenoAgeEAA* | 0.67 (4.75) | -0.74 (4.98) | 0 |
| HEXA | PCHorvath | 0.15 (0.97) | -0.47 (0.94) | 0.75 |
|  | PCHorvathEAA* | 0.02 (0.19) | -0.07 (0.16) | 0 |
|  | PCHannum | 45.47 (6.79) | 47.03 (4.54) | 0.69 |
|  | PCHannumEAA* | 0.45 (4.66) | -1.41 (4.02) | 0 |
|  | PCPhenoAge | 43.6 (7.02) | 46.54 (4.61) | 0.74 |
|  | PCPhenoAgeEAA* | 0.21 (4.63) | -0.64 (4) | 0 |
| * P-value of t-test between male and female < 0.05. a. Data are presented as mean ± standard deviation; b. Pearson correlation coefficient with chronological age; EAA, epigenetic age acceleration; | | | | |

| Table S11. Association between health outcomes and EAAs of epigenetic clocks trained on Japanese data. | | | | | | |
| --- | --- | --- | --- | --- | --- | --- |
|  | Trait | OR (95% CI) /  BETA (SE) | P-value | Trait | OR (95% CI) / BETA (SE) | P-value |
| KARE |  |  |  |  |  |  |
| PCHorvathEAA | T2D | 1 (0.97, 1.02) | 7.85E-01 | Triglyceride | -3.25 (2.67) | 2.24E-01 |
|  | Hypertension | 1 (0.98, 1.03) | 8.38E-01 | hs-CRP | -0.04 (0.11) | 7.02E-01 |
|  | AST | 0.28 (0.36) | 4.36E-01 | FVC % PRED | -0.31 (0.4) | 4.37E-01 |
|  | ALT | 0.07 (0.42) | 8.76E-01 | FEV1% PRED | -0.26 (0.48) | 5.92E-01 |
|  | HDL | 0.14 (0.29) | 6.26E-01 |  |  |  |
| PCHannumEAA | T2D | 1.04 (1.01, 1.06) | 1.84E-03 | Triglyceride | 1.94 (2.51) | 4.40E-01 |
|  | Hypertension | 1.04 (1.01, 1.06) | 1.68E-03 | hs-CRP | -0.09 (0.1) | 3.68E-01 |
|  | AST | 0.62 (0.34) | 6.74E-02 | FVC % PRED | -0.42 (0.38) | 2.66E-01 |
|  | ALT | 0.89 (0.39) | 2.43E-02 | FEV1% PRED | -0.34 (0.45) | 4.50E-01 |
|  | HDL | -0.15 (0.27) | 5.71E-01 |  |  |  |
| PCPhenoAgeEAA | T2D | 1.09 (1.06, 1.11) | 3.93E-10 | Triglyceride | 7.13 (2.96) | 1.61E-02 |
|  | Hypertension | 1.06 (1.03, 1.09) | 4.01E-05 | hs-CRP | 0.28 (0.12) | 2.27E-02 |
|  | AST | 1.43 (0.4) | 3.44E-04 | FVC % PRED | -1.49 (0.44) | 8.11E-04 |
|  | ALT | 1.61 (0.46) | 5.13E-04 | FEV1% PRED | -1.28 (0.54) | 1.70E-02 |
|  | HDL | -0.21 (0.32) | 5.03E-01 |  |  |  |
| HEXA |  |  |  |  |  |  |
| PCHorvathEAA | T2D | 1.02 (0.97, 1.06) | 7.44E-01 | HDL | -0.26 (0.49) | 5.94E-01 |
|  | Hypertension | 1.02 (0.99, 1.06) | 2.52E-01 | Triglyceride | 0.45 (4.73) | 9.24E-01 |
|  | AST | -0.91 (0.71) | 2.00E-01 | hs-CRP | -0.09 (0.05) | 4.67E-02 |
|  | ALT | 0.36 (0.94) | 6.99E-01 |  |  |  |
| PCHannumEAA | T2D | 1.02 (1.01, 1.06) | 3.87E-01 | HDL | -0.68 (0.54) | 2.05E-01 |
|  | Hypertension | 1.03 (0.99, 1.07) | 1.41E-01 | Triglyceride | 4.91 (5.26) | 3.51E-01 |
|  | AST | 2.11 (0.79) | 7.57E-03 | hs-CRP | -0.05 (0.05) | 3.33E-01 |
|  | ALT | 3.52 (1.03) | 6.87E-04 |  |  |  |
| PCPhenoAgeEAA | T2D | 1.07 (1.04, 1.11) | 3.58E-05 | HDL | -0.52 (0.53) | 3.26E-01 |
|  | Hypertension | 1.03 (0.99, 1.07) | 1.29E-01 | Triglyceride | 9.42 (5.16) | 6.84E-02 |
|  | AST | 2.38 (0.77) | 2.14E-03 | hs-CRP | 0.12 (0.05) | 1.46E-02 |
|  | ALT | 3.29 (1.02) | 1.29E-03 |  |  |  |
| All regression models are adjusted for age, sex, and 10 principal components. All EAAs are scaled to mean = 0 and SD = 1. OR, Odds ratios; CI, confidence interval; SE, standard error. | | | | | | |

| Table S12. Association between environmental factors and EAAs of epigenetic clocks trained on Japanese data. | | | | | | |
| --- | --- | --- | --- | --- | --- | --- |
|  | Trait | BETA (SE) | P-value | Trait | BETA (SE) | P-value |
| KARE |  |  |  |  |  |  |
| PCHorvathEAA | BMI | 0.01 (0.01) | 5.63E-02 | Drink | -0.01 (0.02) | 7.04E-01 |
|  | WHR | -0.12 (0.3) | 6.74E-01 | Smoke | 0.02 (0.03) | 6.25E-01 |
|  | Income | 0.02 (0.01) | 1.71E-01 | Pack-years | 0 (0.002) | 7.66E-01 |
|  | Education | 0.03 (0.02) | 1.54E-01 | Exercise | -0.02 (0.04) | 5.72E-01 |
|  | PWI | 0.004 (0.003) | 2.58E-01 |  |  |  |
| PCHannumEAA | BMI | 0.03 (0.01) | 3.04E-06 | Drink | -0.01 (0.03) | 7.71E-01 |
|  | WHR | 0.68 (0.31) | 2.87E-02 | Smoke | 0.05 (0.04) | 1.98E-01 |
|  | Income | 0.01 (0.01) | 5.55E-01 | Pack-years | 0.002 (0.002) | 3.33E-01 |
|  | Education | 0.01 (0.02) | 5.96E-01 | Exercise | 0.01 (0.04) | 7.94E-01 |
|  | PWI | 0.004 (0.004) | 2.97E-01 |  |  |  |
| PCPhenoAgeEAA | BMI | 0.02 (0.01) | 2.52E-03 | Drink | -0.01 (0.02) | 6.76E-01 |
|  | WHR | 1.22 (0.26) | 4.49E-06 | Smoke | 0.11 (0.03) | 1.40E-04 |
|  | Income | -0.01 (0.01) | 1.85E-01 | Pack-years | 0.002 (0.001) | 2.86E-01 |
|  | Education | -0.02 (0.02) | 1.96E-01 | Exercise | -0.02 (0.04) | 5.37E-01 |
|  | PWI | 0.002(0.003) | 5.63E-01 |  |  |  |
| HEXA |  |  |  |  |  |  |
| PCHorvathEAA | BMI | 0.02 (0.01) | 1.30E-02 | PWI | -0.004 (0.004) | 3.36E-01 |
|  | WHR | 1.23 (0.55) | 2.55E-02 | Drink | -0.1 (0.05) | 6.48E-02 |
|  | Income | 0.002 (0.02) | 9.31E-01 | Smoke | -0.14 (0.06) | 2.90E-02 |
|  | Education | -0.02 (0.02) | 4.36E-01 | Exercise | -0.1 (0.09) | 2.58E-01 |
| PCHannumEAA | BMI | 0.03 (0.01) | 1.21E-05 | PWI | -0.006 (0.004) | 9.20E-02 |
|  | WHR | 1.52 (0.49) | 2.11E-03 | Drink | 0.01 (0.05) | 8.36E-01 |
|  | Income | -0.02 (0.02) | 2.99E-01 | Smoke | 0.07 (0.06) | 2.44E-01 |
|  | Education | -0.02 (0.02) | 3.09E-01 | Exercise | -0.22 (0.08) | 5.09E-03 |
| PCPhenoAgeEAA | BMI | 0.01 (0.01) | 4.24E-01 | PWI | -0.002 (0.004) | 6.42E-01 |
|  | WHR | 0.92 (0.5) | 6.58E-02 | Drink | 0.01 (0.05) | 9.15E-01 |
|  | Income | -0.07 (0.02) | 3.32E-04 | Smoke | 0.17 (0.06) | 2.94E-03 |
|  | Education | -0.06 (0.02) | 1.54E-03 | Exercise | -0.2 (0.08) | 1.32E-02 |
| All regression models are adjusted for age, sex, and 10 principal components. All EAAs are scaled to mean = 0 and SD = 1. SE, standard error. | | | | | | |

| Table S13. Cox regression analysis of EAAs for epigenetic clocks trained on Japanese data. | | | | |
| --- | --- | --- | --- | --- |
|  |  |  | HR (95% CI) | P-value |
| KARE | T2D | PCHorvathEAA | 0.8 (0.64, 0.99) | 4.38E-02 |
|  |  | PCHannumEAA | 0.9 (0.74, 1.1) | 3.15E-01 |
|  |  | PCPhenoAgeEAA | 1.12 (0.89, 1.42) | 3.38E-01 |
|  | Hypertension | PCHorvathEAA | 1.05 (0.92, 1.21) | 4.81E-01 |
|  |  | PCHannumEAA | 1.14 (1, 1.3) | 4.93E-02 |
|  |  | PCPhenoAgeEAA | 1.17(1, 1.37) | 4.88E-02 |
| HEXA | Hypertension | PCHorvathEAA | 0.72 (0.48, 1.09) | 1.25E-01 |
|  |  | PCHannumEAA | 0.79 (0.48, 1.31) | 3.62E-01 |
|  |  | PCPhenoAgeEAA | 0.62 (0.38, 1) | 5.02E-02 |
| All Cox models are adjusted for age, sex, and 10 principal components. All EAAs are scaled to mean = 0 and SD = 1. HR, Hazard ratios; CI, confidence interval. | | | | |

FIGURE


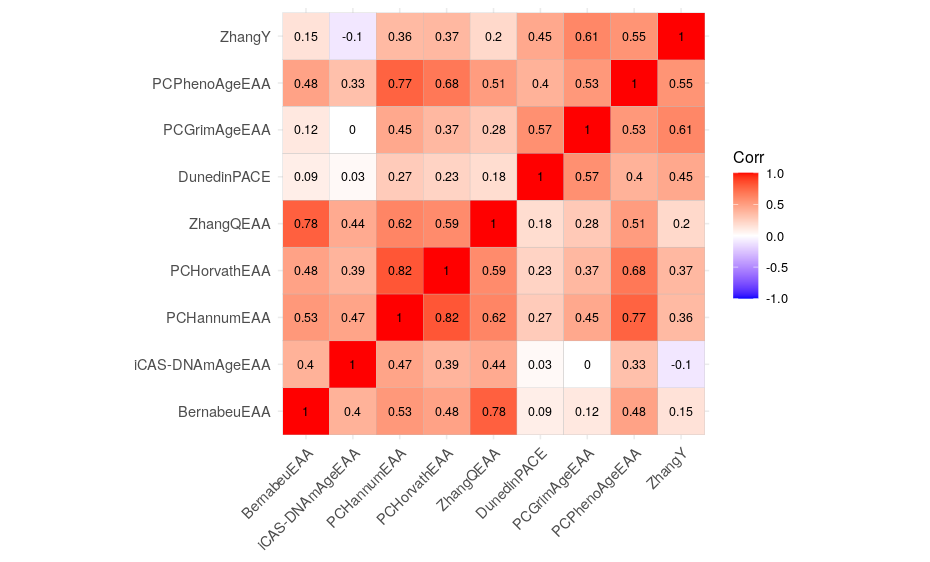


**Figure 1** Correlation matrix of epigenetic age accelerations. Positive correlations are denoted by red shades, with lighter shades signifying weaker correlation values.


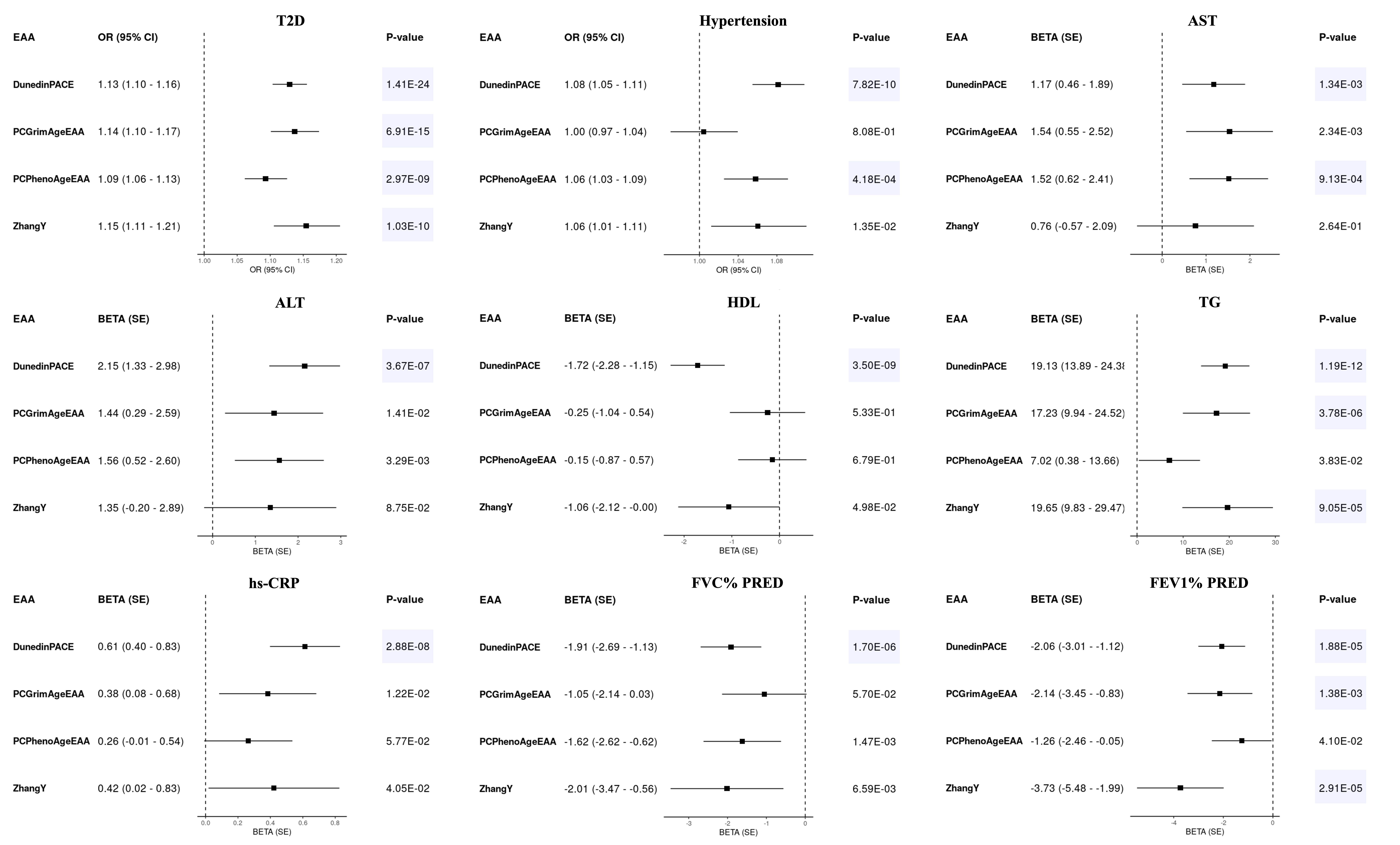


**Figure 2** Forest plots for epigenetic age accelerations (EAAs) of second-generation clocks and health outcomes in KARE. Odds ratios (ORs) with 95% confidence intervals (CIs) or beta values with standard errors (SEs) were displayed along with their corresponding P-values. All regression models adjusted for age, sex, and 10 principal components, with all EAAs scaled to a mean of 0 and a standard deviation of 1. Significant results were highlighted with a blue background based on Bonferroni-corrected P-values (0.05 / (4 × 9) = 1.39E-03).


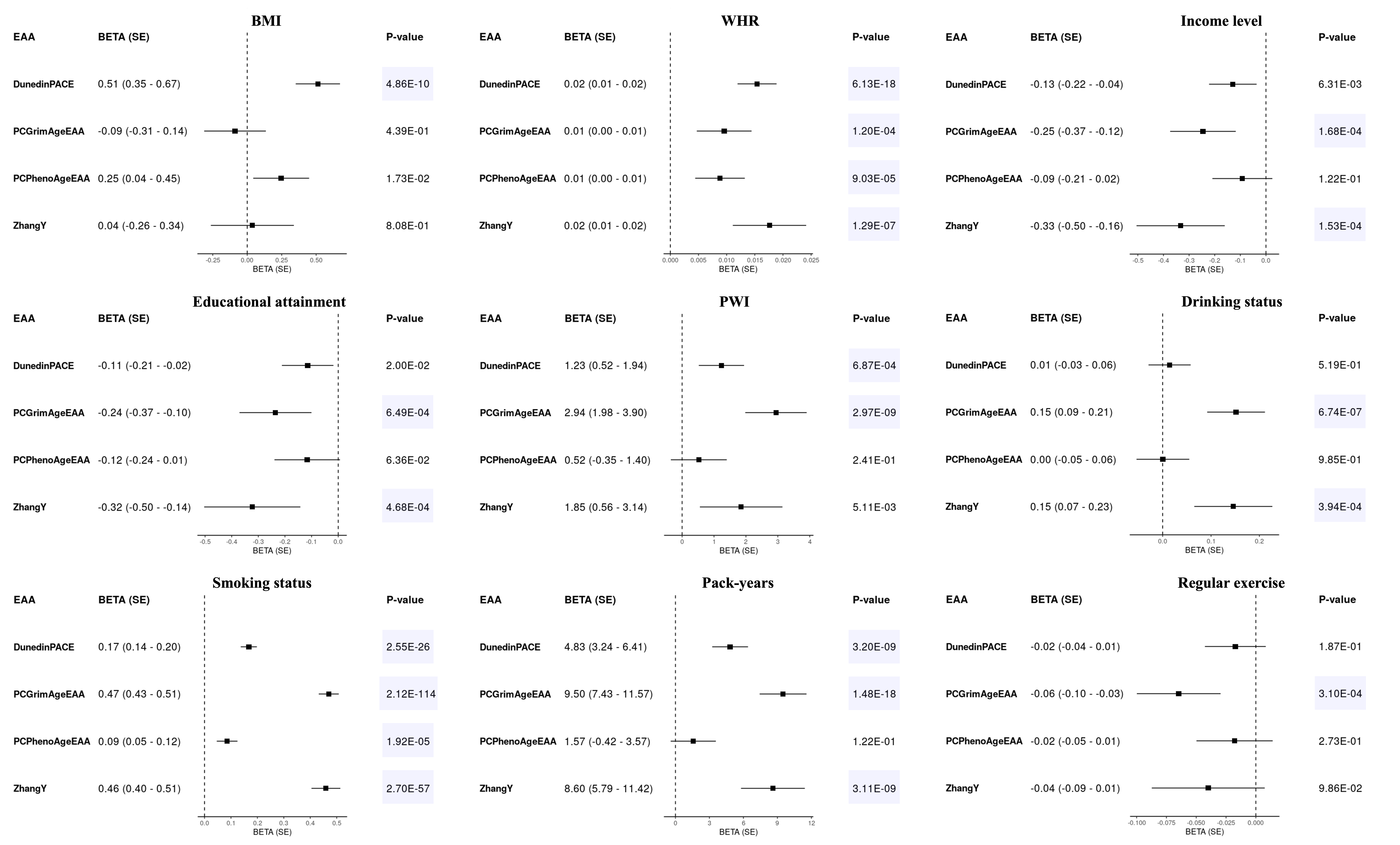


**Figure 3** Forest plots for epigenetic age accelerations (EAAs) of second-generation clocks and environmental factors in KARE. Beta values with standard errors (SEs) were displayed along with their corresponding P-values. All regression models adjusted for age, sex, and 10 principal components, with all EAAs scaled to a mean of 0 and a standard deviation of 1. Significant results were highlighted with a blue background based on Bonferroni-corrected P-values (0.05 / (4 × 9) = 1.39E-03).


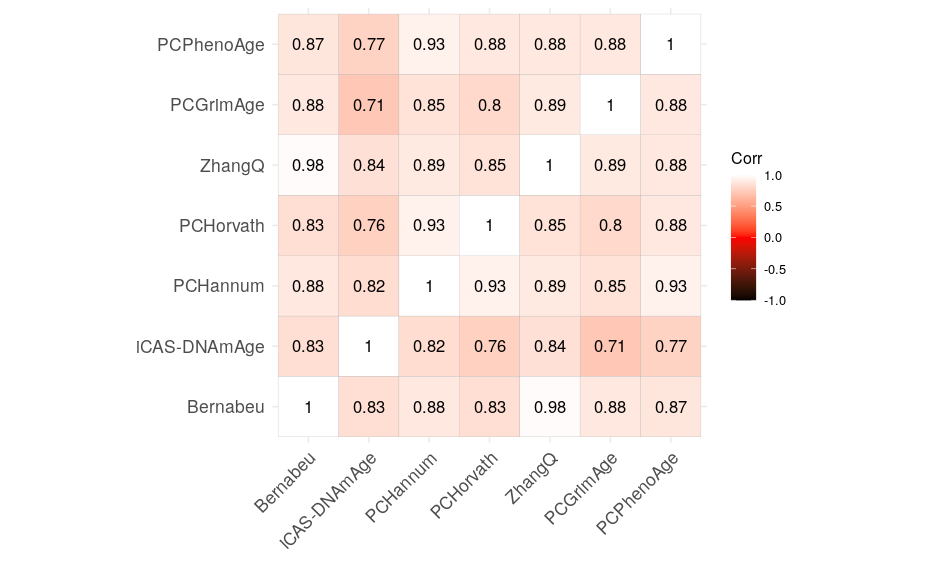


**Figure S1** Correlation matrix of epigenetic clocks. Positive correlations are denoted by white, with darker red shades signifying weaker correlation values.


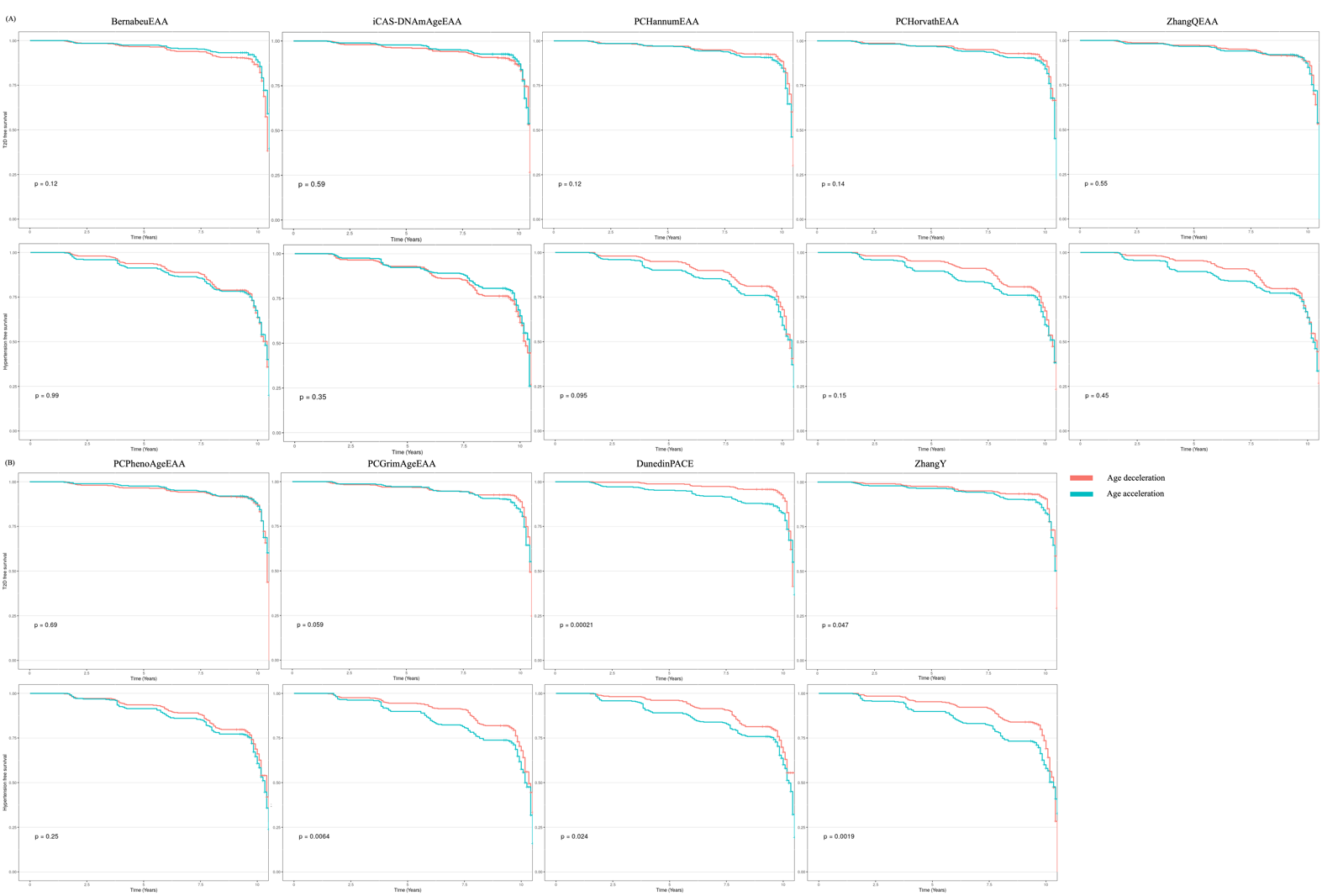


**Figure S2** Karplan-Meier curves for diseases-free survival between epigenetic age acceleration and epigenetic age deceleration in KARE. Figure S2A illustrates first-generation epigenetic clocks, while Figure S2B displays for second-generation epigenetic clocks.


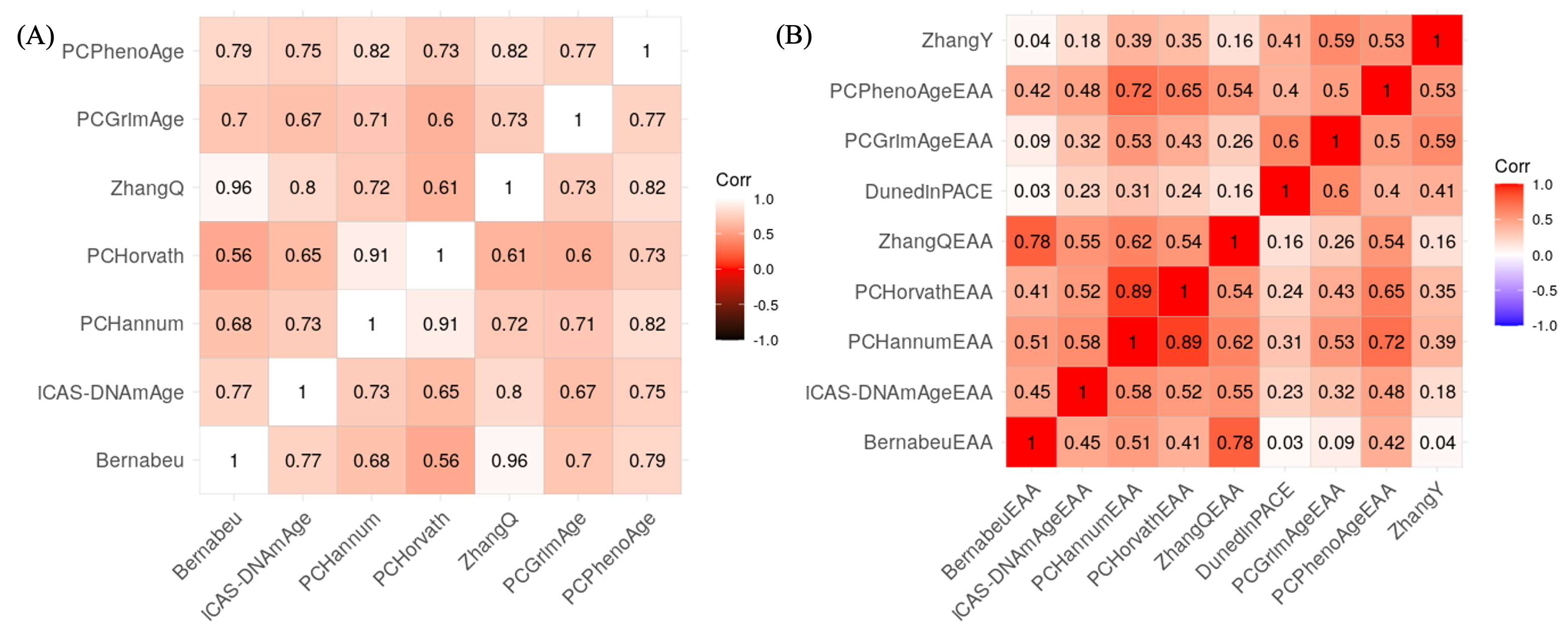


**Figure S3** Correlation matrix of epigenetic clocks and EAAs of epigenetic clocks. Figure S3A illustrates correlation for epigenetic clocks. Positive correlations are denoted by white, with darker red shades signifying weaker correlation values. Figure S3B displays correlation for EAAs of epigenetic clocks. Positive correlations are denoted by red shades, with lighter shades signifying weaker correlation values. The values of DunedinPACE and ZhangY were regarded as EAA.


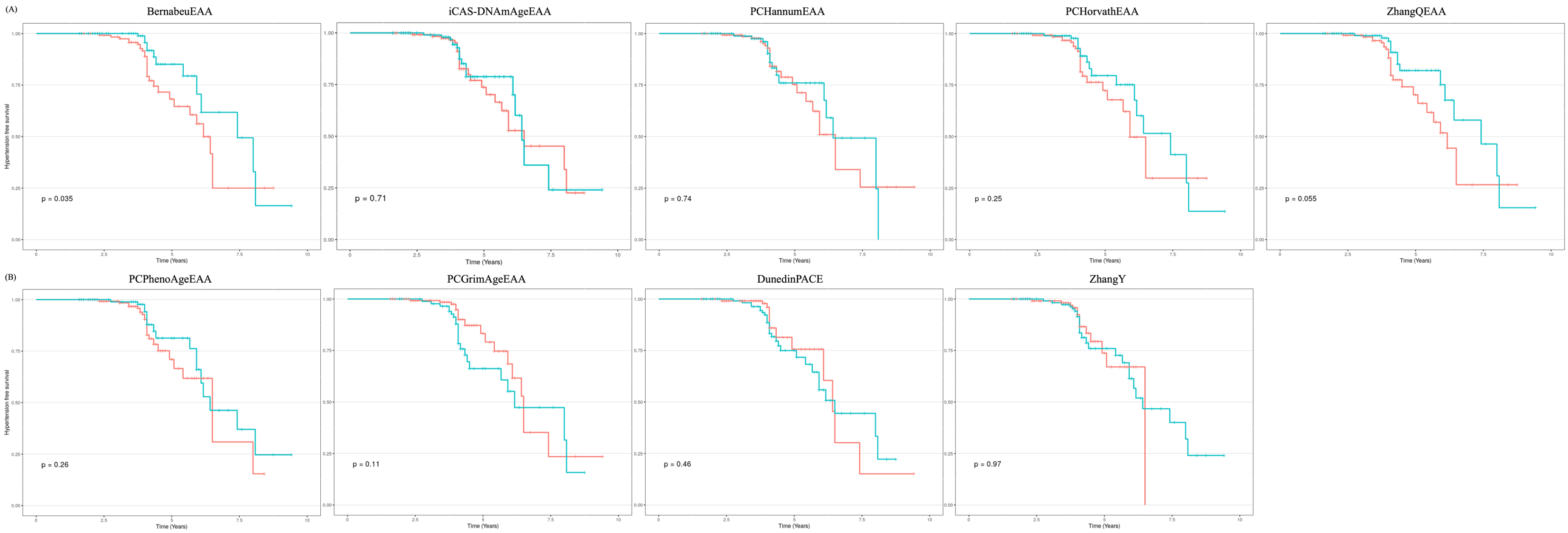


**Figure S4** Karplan-Meier curves for hypertension-free survival between epigenetic age acceleration and epigenetic age deceleration in HEXA. Figure S5A illustrates first-generation epigenetic clocks, while Figure S5B displays for second-generation epigenetic clocks.


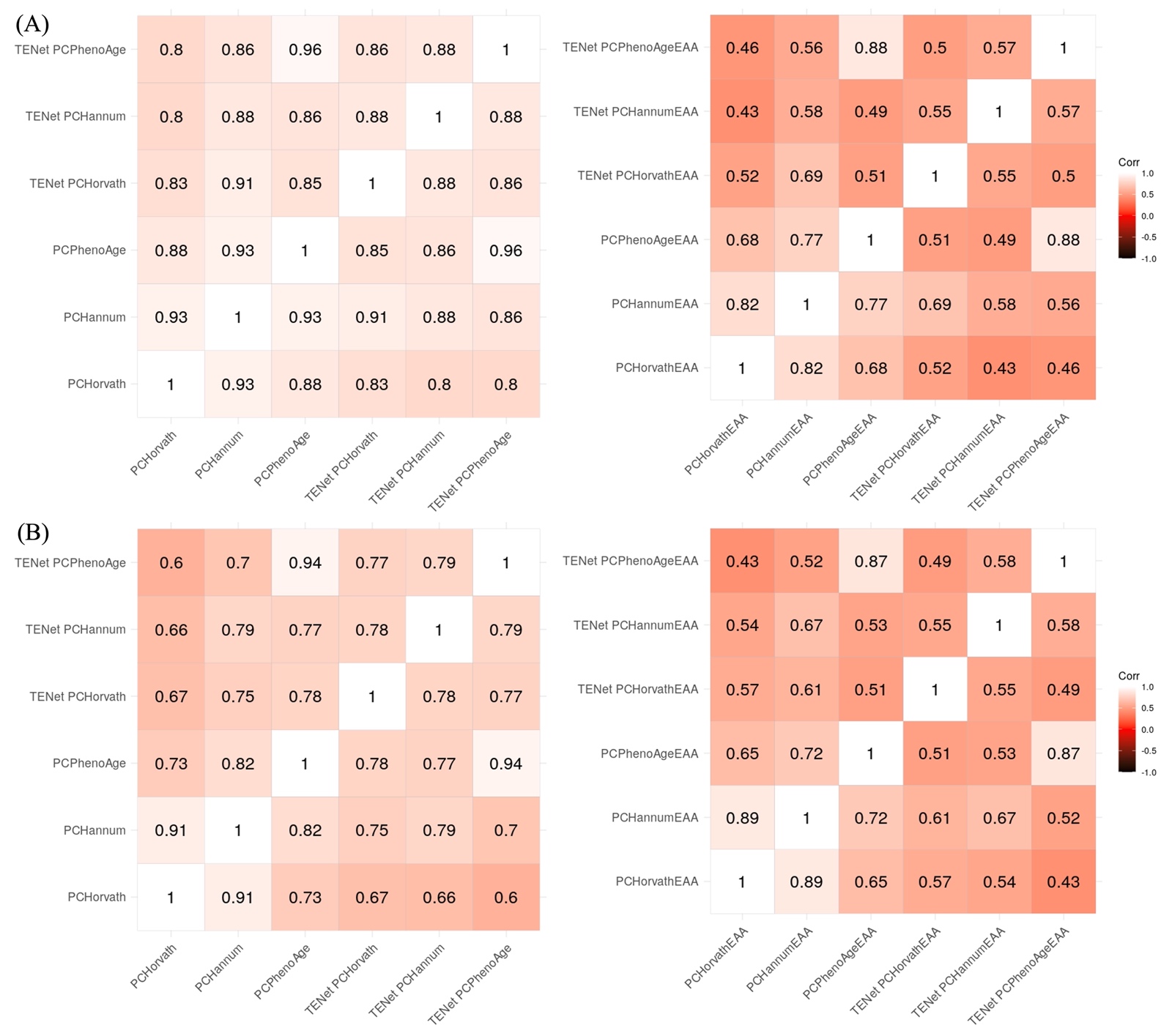


**Figure S5** Correlation matrix of epigenetic clocks and EAAs of epigenetic clocks. Figures A and B show correlations for KARE and HEXA, respectively. Positive correlations are denoted by white, with darker red shades signifying weaker correlation values. TENet mean was trained using Transfer Elastic Net on Japanese data.


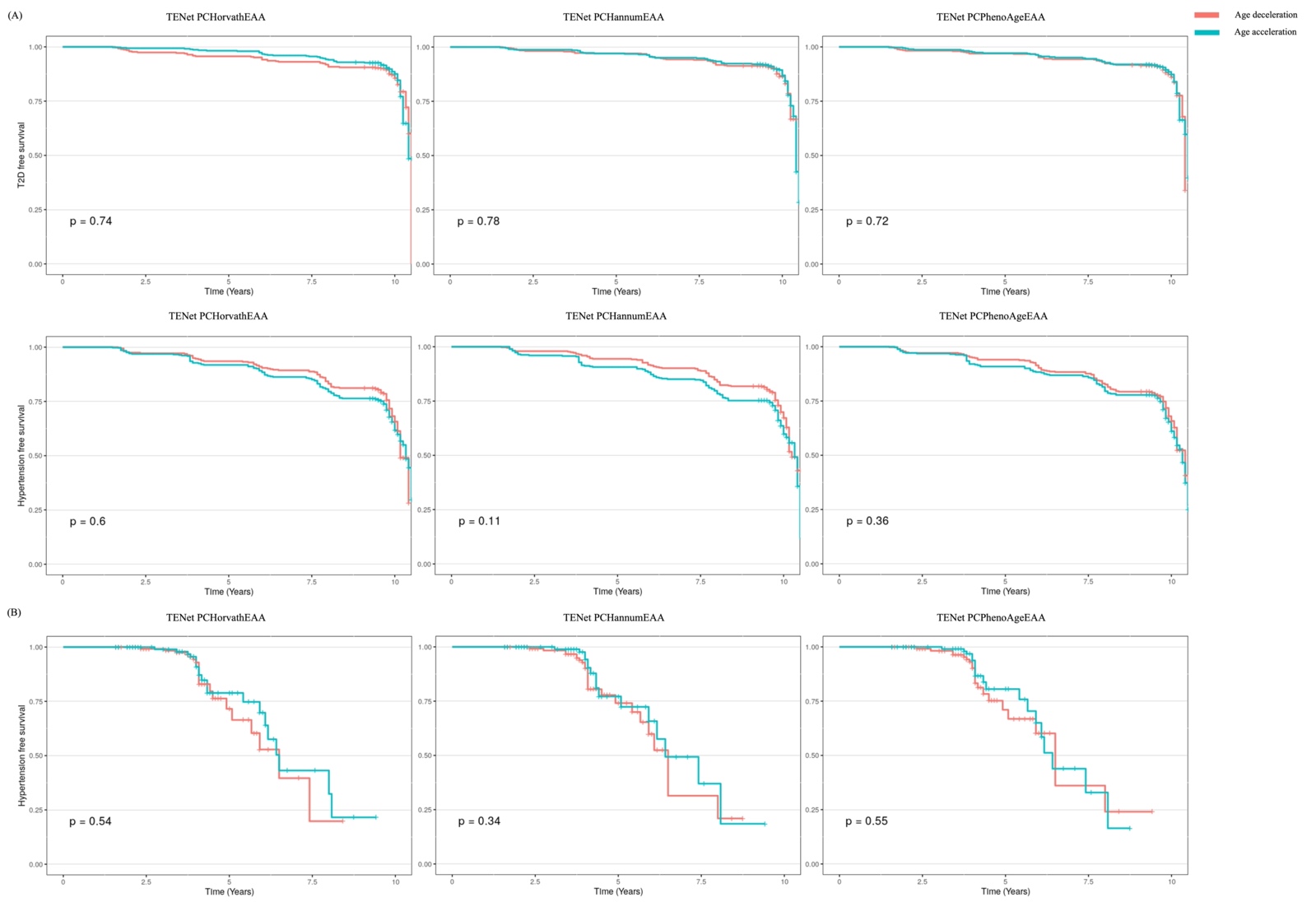


**Figure S6** Karplan-Meier curves for disease-free survival between epigenetic age acceleration and epigenetic age deceleration. Figures A and B illustrate the results for KARE and HEXA, respectively. TENet mean was trained using Transfer Elastic Net on Japanese data.


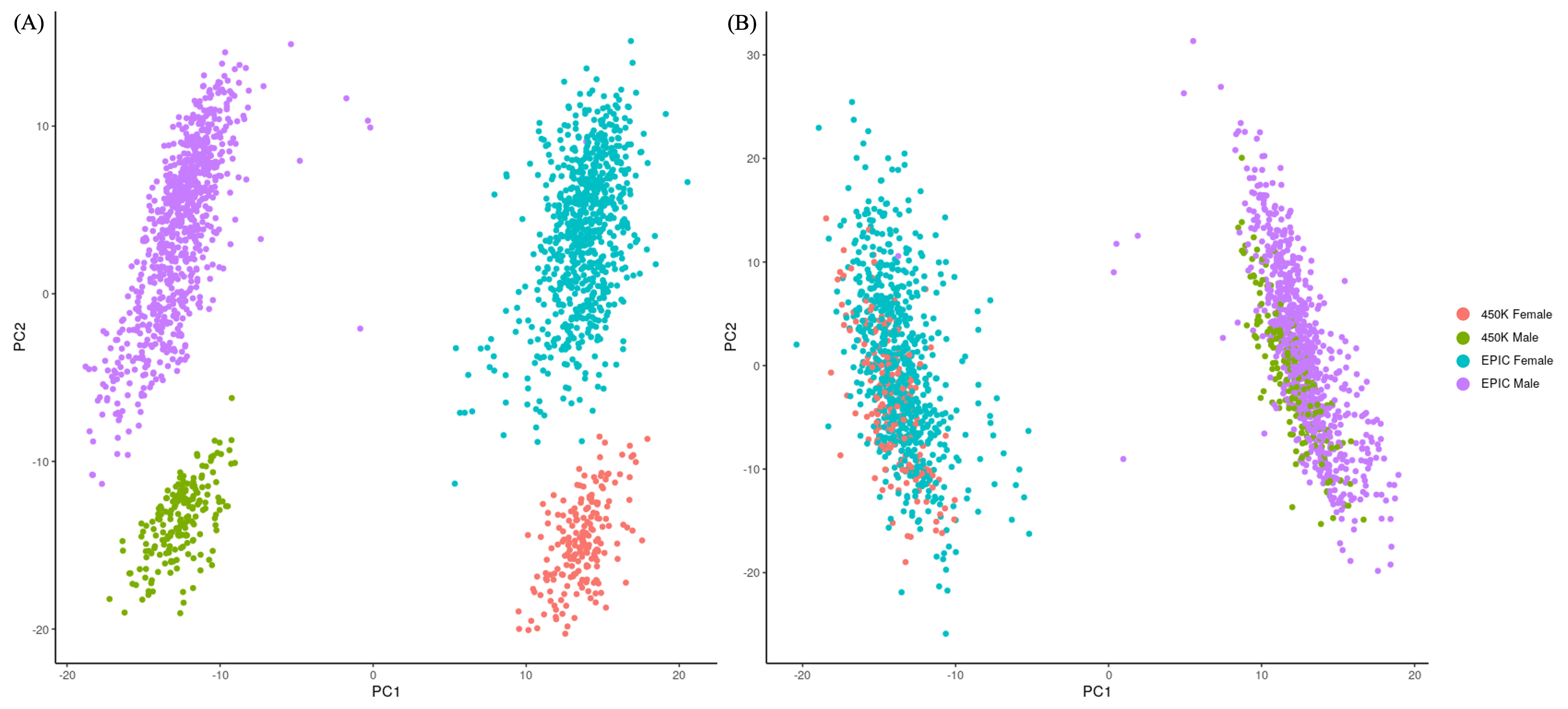


**Figure S7** PCA plots of KARE. Figures A and B illustrate the principal components of methylation data before and after batch correction.


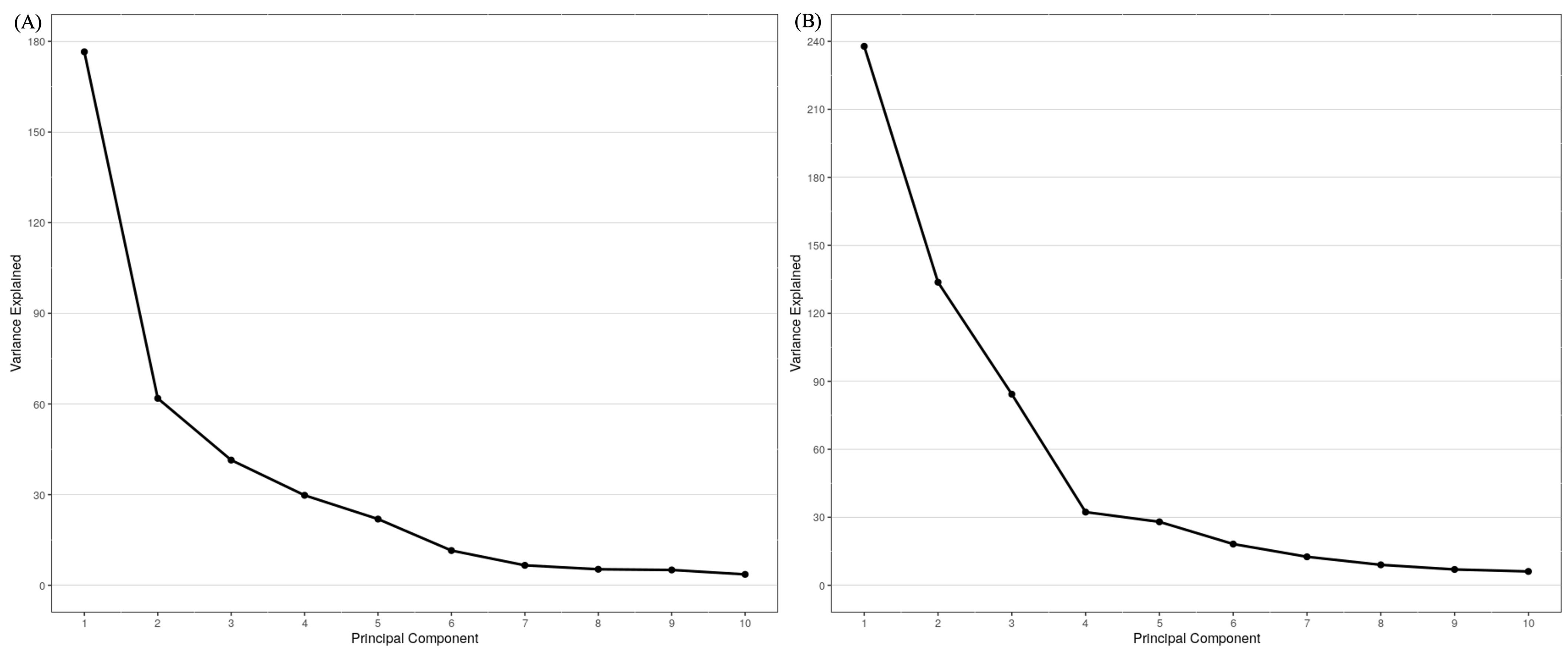


**Figure S8** Elbow curves of the principal component variances for KARE (A) and HEXA (B).
